# Examining the Reversibility of Long-Term Behavioral Disruptions in Progeny of Maternal SSRI Exposure

**DOI:** 10.1523/ENEURO.0120-18.2018

**Published:** 2018-07-09

**Authors:** Susan E. Maloney, Shyam Akula, Michael A. Rieger, Katherine B. McCullough, Krystal Chandler, Adrian M. Corbett, Audrey E. McGowin, Joseph D. Dougherty

**Affiliations:** 1 Department of Genetics, Washington University School of Medicine, St. Louis, MO 63110; 2 Department of Psychiatry, Washington University School of Medicine, St. Louis, MO 63110; 3 Department of Neuroscience, Cell Biology and Physiology, Wright State University, Dayton, OH 45435; 4 Department of Chemistry, Wright State University, Dayton, OH 45435

**Keywords:** autism, fluoxetine, sensory sensitivity, serotonin, social behavior, SSRI

## Abstract

Serotonergic dysregulation is implicated in numerous psychiatric disorders. Serotonin plays widespread trophic roles during neurodevelopment; thus perturbations to this system during development may increase risk for neurodevelopmental disorders. Epidemiological studies have examined association between selective serotonin reuptake inhibitor (SSRI) treatment during pregnancy and increased autism spectrum disorder (ASD) risk in offspring. It is unclear from these studies whether ASD susceptibility is purely related to maternal psychiatric diagnosis, or if treatment poses additional risk. We sought to determine whether maternal SSRI treatment alone or in combination with genetically vulnerable background was sufficient to induce offspring behavior disruptions relevant to ASD. We exposed C57BL/6J or *Celf6* ^+/-^ mouse dams to fluoxetine (FLX) during different periods of gestation and lactation and characterized offspring on tasks assessing social communicative interaction and repetitive behavior patterns including sensory sensitivities. We demonstrate robust reductions in pup ultrasonic vocalizations (USVs) and alterations in social hierarchy behaviors, as well as perseverative behaviors and tactile hypersensitivity. *Celf6* mutant mice demonstrate social communicative deficits and perseverative behaviors, without further interaction with FLX. FLX re-exposure in adulthood ameliorates the tactile hypersensitivity yet exacerbates the dominance phenotype. This suggests acute deficiencies in serotonin levels likely underlie the abnormal responses to sensory stimuli, while the social alterations are instead due to altered development of social circuits. These findings indicate maternal FLX treatment, independent of maternal stress, can induce behavioral disruptions in mammalian offspring, thus contributing to our understanding of the developmental role of the serotonin system and the possible risks to offspring of SSRI treatment during pregnancy.

## Visual Abstract

**Figure:**
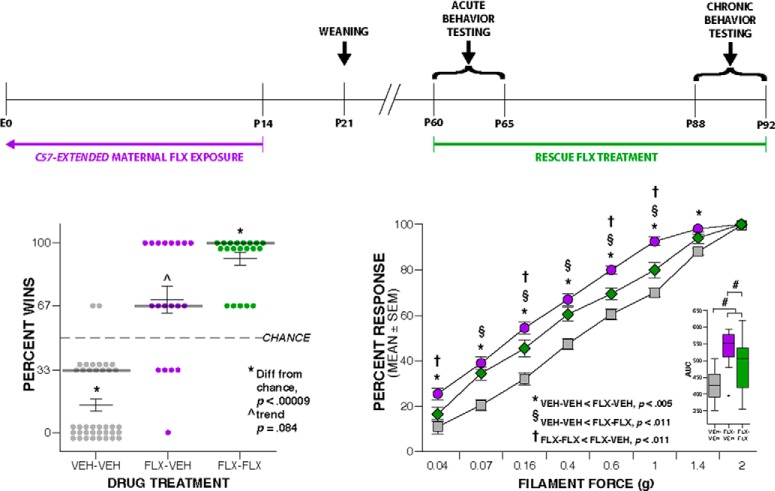

## Significance Statement

Human epidemiological studies suggest that taking antidepressants during pregnancy may increase risk autism spectrum disorder (ASD) in offspring. Since only women with a diagnosis take antidepressants, there is substantial debate on whether all increased ASD risk is contributed by the diagnosis, or if medication has an additional influence. We reasoned empirical studies in a reduced system might provide some indication if there was biological basis for such an influence. Our mouse studies show that, in the absence of other maternal manipulations or stressors, maternal selective serotonin reuptake inhibitor (SSRI) exposure alone can alter the behavioral circuits for sensory, social, and repetitive behaviors, relevant to ASD, in a mammalian brain, and that some of these changes are reversible by SSRI re-exposure.

## Introduction

Dysregulation of the serotonin (5-hydroxytryptamine; 5-HT) system is implicated in numerous psychiatric disorders (Nordquist and Oreland, 2010). This system innervates the entire CNS, allowing 5-HT to influence a variety of behavioral functions including: sleep-wake cycle, perception, appetite, aggression, sexual behavior, sensorimotor activity, pain sensitivity, mood, and learning and memory (Lucki, 1998; Smythies, 2005). During prenatal development, 5-HT is one of the earliest neuromodulators to become active, and 5-HT levels, the expression of the 5-HT transporter, and 5-HT receptors are at their peak, allowing 5-HT to modulate critical neurodevelopmental processes such as neurogenesis, neuroapoptosis, dendritic refinement, cell migration, and synaptic plasticity (Sodhi and Sanders-Bush, 2004; Whitaker-Azmitia, 2010). During this time, the placenta is a transient source of 5-HT for the fetal forebrain until the forebrain is innervated by 5-HT-producing raphe fibers (Muller et al., 2016). Increased 5-HT transfer from the placenta has been shown to blunt 5-HT axonal outgrowth within the fetal forebrain (Goeden et al., 2016). Thus, alterations to 5-HT activity from either exogenous maternal or endogenous fetal sources can impact circuit development, possibly increasing risk for psychiatric disorders.

5-HT is a dominant target for treatment in many psychiatric conditions through frequently prescribed medications such as selective serotonin reuptake inhibitors (SSRIs). SSRIs have become the first-line pharmacotherapy for mood disorders in pregnant women (Andrade et al., 2008) and are among the most commonly prescribed medications in this population, with frequency estimates in the United States at 5–13% (Cooper et al., 2007; Andrade et al., 2008; Ramos et al., 2008). As the number of pregnant women taking antidepressants has increased, so has the number of studies investigating their safety and effects during pregnancy. Initial studies on neonatal outcomes reported no gross abnormalities (Misri et al., 2000); however, adverse outcomes like low birth weight and respiratory distress were reported (Oberlander et al., 2006). Sufficient time has accrued since SSRIs were introduced that human epidemiological studies are now able to assess the impact of SSRI use during pregnancy on risk of offspring psychiatric disorder diagnoses. The initial focus has been on autism spectrum disorder (ASD), likely because of the young age at onset and because 5-HT dysregulation has been implicated in ASD: 30% of ASD patients exhibit elevated 5-HT levels in whole-blood platelets (Benza and Chugani, 2015), changes to 5-HT can either worsen or alleviate certain symptoms (McDougle et al., 1993, 1996; Hollander et al., 2005), increased 5-HT axons are observed postmortem (Azmitia et al., 2011), and PET studies demonstrate altered 5-HT synthesis *in vivo* (Chugani et al., 1999, 1997). A meta-analysis of the recent epidemiological studies examining this possible SSRI-ASD link reported a significant case-control association between maternal antidepressant use and ASD risk in offspring. This remained when adjusted for maternal psychiatric history (Odds Ratio [OR], 1.52; 95% Confidence Intervals [CI], 1.09–2.12; Mezzacappa et al., 2017), although parallel analysis of existing cohort studies did not quite show independence from psychiatric history (HR, 1.26; 95%CI, 0.91–1.74). Likewise, two additional studies provide evidence supporting (OR 1.45; 95%CI, 1.13–1.85; Rai et al., 2017), and not clearly supporting (OR 1.23; 95%CI 0.96–1.57; Viktorin et al., 2017), an effect of antidepressant usage independent from maternal diagnosis. Thus, although inconsistent in rejecting the null hypothesis, the CIs reported also clearly do not reject a modest independent effect of magnitude on par or above that typically seen for common genetic variants in psychiatric disease (ORs ∼1.1; Schizophrenia Working Group of the Psychiatric Genomics Consortium, 2014). Regardless, direct causality and biological mechanisms cannot be inferred from epidemiological studies. However, animal studies can provide clear indication as to whether transient SSRI exposure, independent of maternal psychiatric stress, can alter long-term behaviors in mammals and provide ready access to related neurobiology.

We developed a rodent model of maternal SSRI exposure, in the absence of maternal stress, to determine whether drug alone induces behavioral disruptions related to the core symptoms of ASD in offspring. As genetic factors are clearly an important causation of ASD (Geschwind, 2008), it is likely that environmental contributions to ASD risk interact with existing genetic susceptibility (Hertz-Picciotto et al., 2006; Klei et al., 2012). It has been suggested that environmental factors that might modulate social behavior or language could tip the balance toward ASD in children with genetic vulnerability (Geschwind, 2008). As we initially thought SSRI exposure alone might be a relatively modest factor, we also exposed *Celf6* mutant mice, which exhibit a subtle ASD-like phenotype (Dougherty et al., 2013), to maternal SSRI and analyzed offspring behavior for possible potentiation of the ASD-like phenotype. The *Celf6* mutant was ideal for this gene × environment experiment because this model already shows subtle ASD-related deficits, specifically decreased early social communicative behavior and a resistance to change behavior patterns (Dougherty et al., 2013), which allows for possible further disruption to other social and repetitive behaviors with the addition of FLX. Further, Celf6 is enriched in 5-HT-producing cells and, when deleted, results in a decrease in brain 5-HT levels (Dougherty et al., 2013). Thus, we hypothesized that early exposure to FLX may interact synergistically on the 5-HT system to further disrupt behavior in mice with this genetically vulnerable background. We also examined the impact of adult SSRI re-exposure on ameliorating these disruptions to better understand their mechanism: if persistent alterations in 5-HT activity levels are playing a key role in these behavioral abnormalities, pharmacotherapy should reverse them. If not, it would indicate underlying behavioral circuits were permanently altered by maternal SSRI exposure. Overall, across multiple exposure durations, we found strong evidence supporting the hypothesis that transient exposure to SSRIs has long-term consequences on behaviors relevant to ASD symptoms. Furthermore, while a subset of these consequences are reversible with acute or chronic adult SSRI re-exposure, other phenotypes are exacerbated. Thus, maternal SSRI exposure has complex, long-lasting effects on the serotonergic system in the mammalian brain.

## Materials and Methods

### Animals

All animal procedures were performed in accordance with the Washington University in St. Louis animal care committee regulations. Mice were house in translucent plastic cages measuring 28.5 × 17.5 × 12 cm with corncob bedding and standard lab diet and water freely available. The colony room lighting was a 12/12 h light/dark cycle; room temperature (∼20–22°C) and relative humidity (50%) were controlled automatically. All mice used in this study were maintained and bred in the vivarium at Washington University in St. Louis and were all group-housed. The C57BL/6J wild-type (WT) inbred strain (https://www.jax.org/strain/000664; RRID: IMSR_JAX:000664) and the *Celf6* mutant line (https://www.jax.org/strain/028389; RRID: IMSR_JAX:028389) were used in this study. Five separate cohorts of mice were used based on maternal drug exposure duration and mouse line: *Celf6*-Extended, C57-Extended, Long Prenatal, Short Prenatal, and Rescue (Table 1). *Celf6* mutant mice were generated on the C57BL/6 background by deletion of exon 4 of the *Celf6* gene as previously described (Dougherty et al., 2013). For the *Celf6*-Extended cohort, heterozygous breedings pairs were used to generate *Celf6*^+/+^, *Celf6*^+/-^, and *Celf6*^-/-^ littermates (Table 1). Offspring were genotyped using standard reagents and primers for amplification of the region spanning exons 3 and 4: forward, ATCGTCCGATCCAAGTGAAGC and reverse, CTCCTCGATATGGCCGAAGG. C57BL/6J breeding pairs were used to generate the C57-Extended, Long Prenatal, Short Prenatal, and Rescue cohorts (Table 1). The C57-Extended cohort served to replicate and extend the findings from the *Celf6*-Extended cohort. Mice were examined for ultrasonic vocalization (USV) production, developmental milestones, and reflexes, and subsets were used for further behavioral assessment.

**Table 1.** Cohort sample sizes distributed between sexes, and behavioral tests

		FLX exposed				Vehicle exposed				
Cohort	Genotype	Males	Females	Total	Litters	Males	Females	Total	Litters	Behavioral tests
*Celf6*-Extended	*Celf6* ^+/+^	11	12	23		10	9	19		Developmental milestones and reflexes, sensorimotor battery, social approach, marble burying, T-maze, 1-h locomotor activity
	*Celf6* ^+/-^	33	23	56	19	32	19	51	17	
	*Celf6* ^-/-^	13	12	25		15	14	29		
C57-Extended	C57BL/6J	14	16	30	4	16	19	35	5	Juvenile play, marble burying, T-maze, tube test, von Frey assessment
Long Prenatal	C57BL/6J	7	13	20	3	16	9	25	4	Developmental milestones and reflexes, social approach, T-maze, marble burying, tube test
Short Prenatal	C57BL/6J	10	13	23	4	9	13	22	3	
Rescue	C57BL/6J	9 + VEH	10 + VEH	19	9	19 + VEH	20 + VEH	39	7	von Frey assessment, T-maze, tube test, 1-h locomotor activity
		10 + FLX	10 + FLX	20						

### Maternal SSRI exposure

**Figure 1.**
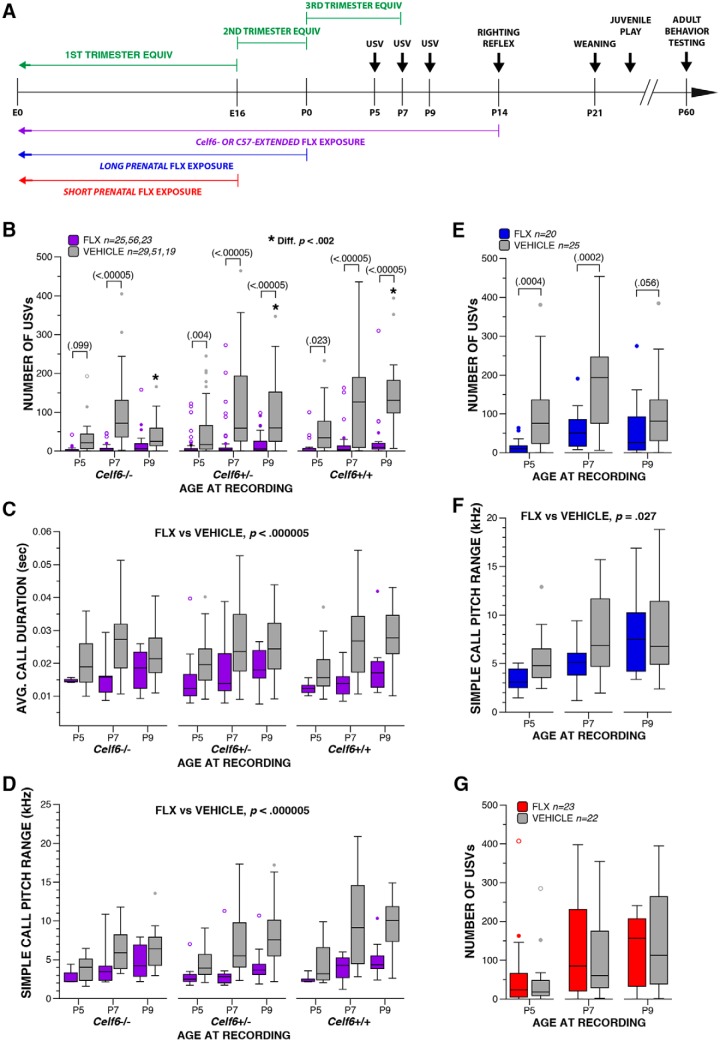
Maternal FLX throughout pregnancy alters early communicative behavior. ***A***, Schematic of the paradigm for maternal FLX exposure, with approximate equivalents in brain development to human pregnancy, and the mouse age for each behavioral test. ***B***, Boxplot of number of USVs at P5, P7, and P9 from *Celf6*-Extended FLX and VEH *Celf6* mutant and WT littermates (drug, *p* < 0.000005; age × drug × genotype interaction, *p* = 0.049); * denotes significant difference at *p* < 0.002 between P9 VEH-exposed *Celf6* mutant and WT littermates. ***C***, ***D***, Boxplots of number average USV duration (***C***; drug, *p* < 0.000005) and pitch range of simple USV calls (***D***; drug, *p* < 0.000005) at P5, P7, and P9 from *Celf6*-Extended FLX and VEH *Celf6* mutant and WT littermates. ***E***, Boxplot of number of USVs at P5, P7, and P9 from Long Prenatal FLX and VEH mice (drug, *p* = 0.0001). ***F***, Boxplot of pitch range of simple USV calls from Long Prenatal FLX and VEH pups (drug, *p* = 0.027). ***G***, Boxplot of number of USVs at P5, P7, and P9 from Short Prenatal FLX and VEH mice (drug, *p* = 0.840). For boxplots, thick horizontal lines signify respective group medians, boxes are 25th–75th percentiles, whiskers are 1.5 × IQR, closed and open circles depict outliers.

In most countries, fluoxetine (FLX, Prozac) was the first SSRI to become available for clinical use (Hiemke and Härtter, 2000). Therefore, FLX is likely to be the most-represented antidepressant in the epidemiological studies of SSRI use during pregnancy. To mimic the 5-HT system in human mothers already taking an antidepressant before pregnancy, dams were exposed to FLX at least one week before mating. FLX crosses the placental barrier at a rate in mice comparable to that in humans (Noorlander et al., 2008). To avoid inducing unwanted maternal stress that can occur with daily injections, which has been shown to have adverse effects on the developing brain (Matrisciano et al., 2013), FLX was administered orally through drinking water sweetened with 1% saccharin to mask unpleasant drug taste. Control dams received 1% saccharin-only water (VEH). FLX capsules (20 mg each; Camber Pharmaceuticals, Inc) were dissolved into water containing 1% saccharin sodium salt hydrate (Millipore Sigma). The FLX dose used in this study was equivalent to the maximum recommended human dose (MRHD) of 80 mg/d on a mg/m^2^ basis (Marken and Munro, 2000). The dose calculations are based on equivalent surface area dosage conversion factors (Freireich et al., 1966) and approximate drinking water consumed daily (Bachmanov et al., 2002). Average drug water intake per day was recorded throughout the study to monitor drug exposure levels. The FLX water was prepared so that each mouse would consume 48 mg/d (16 mg/kg/d based on a 30-g mouse) or 6.5 ml/d of 0.074 mg/ml FLX in 1% saccharin water. Females of the same drug group were co-housed to reduce stress induced by isolation housing, and placed into the cage of a single-housed male for breeding. On detection of a vaginal plug following breeding, the females were removed from the male to isolate maternal drug exposure effects and avoid paternal drug exposure. Three drug exposure durations were used. Extended exposure continued until postnatal day (P)14, the age just before pups begin to consume food and water, to avoid direct drug exposure in the pups. Long Prenatal exposure lasted until birth of the pups, and Short Prenatal exposure was stopped at embryonic day (E)16 (Fig. 1*A*).

### Adult SSRI re-exposure

At P60, FLX or VEH was administered orally through drinking water sweetened with 1% saccharin. All parameters and dosing were as described above. Average drug water intake per day was recorded throughout the study to monitor drug exposure levels.

### HPLC

Reverse-phase HPLC with fluorescence detection was used to separate and quantify FLX and its major active metabolite norfluoxetine (NFLX) in mouse brain tissue according to previously published methods (Unceta et al., 2010; Corbett et al., 2012). P9 mouse pups and adult dams exposed to extended FLX or VEH were deeply anesthetized via isoflurane, killed via rapid decapitation, and the brain extracted and flash frozen in –70° isopentane and stored at –80˚C until HPLC preparation.

#### Reagents and materials

Fluoxetine hydrochloride (FLX; lot #SLBL4347V) and its primary active metabolite, norfluoxetine hydrochloride (NFLX), were purchased from Sigma-Aldrich. Sodium acetate buffer (0.050 M) was prepared from sodium acetate (Fisher Scientific, Inc.) and glacial acetic acid (VWR brand). Borate buffer (0.1 M) was prepared from boric acid, H_3_BO_4_ (Sigma) and sodium hydroxide (Fisher Scientific). Solvents were HPLC-grade acetonitrile (Pierce) and water purified using a Milli-Q system (Millipore Corporation). Stir bar sorptive extraction (SBSE) was performed using GERSTEL-Twister sorptive stir bars (GERSTEL Gmbh & Co. KG) obtained from Agilent Technologies. The stir bars are 10-mm long and are coated with a 0.5-mm film thickness of polydimethylsiloxane (PDMS). Extractions were conducted in Fisherbrand 21 × 70-mm amber glass vials. Desorptions were performed in Varian 4.0-ml clear glass vials with PTFE/sil septa containing Agilent 400-µl glass inserts.

#### Sample preparation

Approximately 100-mg samples of brain tissue (±0.1 mg) were weighed. One milliliter of purified water was added to each sample before homogenization. Four control samples were spoked with FLX and NFLX to yield a final concentration of 120 and 150 ng of FLX and NFLX, respectively.

#### Instrumentation

Chromatographic separations were conducted on a Varian ProStar HPLC system with Galaxie software, a Varian ProStar Model 410 autosampler, and a Hitachi Model L-2485 Elite LaChrom fluorescence detector. The fluorescence detector was set at 228 nm (excitation) and 284 nm (emission). Separations of 100-µl injections were achieved on a GRACE Platinum C18 reverse-phase column (250 × 4.6 mm, 5-µm particle size). The mobile phase consisted on a 30:70 (v:v) of 0.050 M sodium acetate buffer (pH 4.5) and acetonitrile delivered isocratically at a flow rate of 1.0 ml/min. The retention times for NFL and FLX were 10.0–10.9 and 11.7–12.0 min, respectively.

#### Method validation

Individual stock solutions were prepared of 160 mg/l of FLX and 200 mg/l of NFLX in acetonitrile by weighing 1–2 mg of each solid standard to a 10.00 ml and diluting with acetonitrile. The solutions were stored in the freezer at –20°C. A mixed stock solution of FLX and NFLX was prepared in acetonitrile by combining 5 mL of each individual stock solution in to a vial for a final concentration of 80 and 100 µg/ml, respectively, and stored in the dark at –20°C. Calibration standard solutions were prepared in acetonitrile and ranged from 0.016 to 10 µg/ml. Calibration curves were linear over the entire range of calibration with *R* ^2^ for FLX and NFLX ranging from 0.9998 to 0.9999. The limit of detection for FLX was 16 parts-per-billion (ppb) and for NFLX was 20-ppb concentration in solution. When calculated as tissue concentration and corrected for recovery, the limits of detection were 164 ng/g for FLX and 320 ng/g for NFLX.

#### SBSE of FLX and NFLX

Before use, each Gerstel stir bar was washed with acetonitrile for 20 min in a 15-ml vial with the magnetic stirrer set at 300 rpm at 75°C, rinsed with purified water, and patted dry with a lint-free tissue. One mL of 0.1 M borate buffer was added to each brain tissue sample and a stir bar was added. Each sample was stirred at 300 rpm at 75°C for 45 min and allowed to cool to room temperature. The stir bar was removed with a magnet on the outside of the extraction vial. The stir bar was rinsed with purified water and patted dry with a lint-free tissue. For desorption, the stir bar was placed into a 2-ml sample vial with a glass vial-insert into which 0.350-ml acetonitrile had been added. Vials were capped and the analytes desorbed by magnetic agitation at 300 rpm and at 75°C for 30 min. Each vial was cooled slightly before opening to remove the stir bar with a magnet on the outside of the vial. The vial caps were replaced and the samples analyzed.

### Behavioral tasks

Multiple behavioral assays across the same domain were employed to adequately determine presence of behavioral disruptions. Experimenters were blinded to experimental group designations during behavioral testing. Experimenters were all female, except during *Celf6*-Extended developmental assessments in which one female and one male experimenter each collected data. No effect of experimenter sex was observed for those data. Order of and age at testing were chosen to minimize effects of stress and previous testing. Developmental reflexes and milestones assessment of the *Celf6*-Extended, Long Prenatal, and Short Prenatal cohorts occurred on P5–P14. Adult behavioral testing for all cohorts began at P60. Adult behavioral testing of the *Celf6*-Extended cohort included a battery of sensorimotor measures, followed by the social approach test, marble burying, spontaneous alternation T-maze, and the 1-h locomotor activity task. Mice in the C57-Extended cohort were assessed in the juvenile interaction task P22–P30, and adult behavioral testing included marble burying, spontaneous alternation T-maze, the tube test of social dominance, and the von Frey assessment of tactile sensitivity. Both the Long Prenatal and Short Prenatal cohorts were tested as adults in the social approach test, followed by spontaneous alternation T-maze, marble burying, and the tube test of social dominance. Following initiation of FLX or VEH re-exposure at P60, mice in the Rescue cohort were immediately tested for tactile sensitivity in the von Frey assessment, spontaneous alternation T-maze, tube test of social dominance, and the 1-h locomotor activity test to assess acute effects of re-exposure. After three weeks of re-exposure, all mice were retested in the same tasks to assess chronic effects of re-exposure on behavior. The Rescue cohort was not tested before re-exposure, such that no testing occurred during the pre-weaning period or juvenile development.

#### Maternal isolation-induced USV recording

USVs are considered a strongly conserved affective and communicative display that elicits maternal search and retrieval responses, nursing, and caretaking, and is used in the rodent literature to model early communicative deficits (Haack et al., 1983). Playback experiments demonstrated lactating dams respond rapidly with searching behavior to pup isolation calls. In addition, these dam behaviors are dependent on acoustic call features, such as duration and frequency, suggesting these features have communicative value (Wöhr et al., 2008). This behavior has a distinct developmental trajectory, allowing its use for the study of both early communication and neurobehavioral development in infant rodents (Branchi et al., 2001). USV production due to maternal isolation in the C57BL/6J mouse pup normally peaks just after P7, disappearing completely by P14 (Rieger and Dougherty, 2016).

For this study, USV recording occurred on P5, P7, and P9. Dams were removed from the home cage and placed into a clean standard mouse cage for the duration of testing. Pups in the home cage were placed into a warming box (Harvard Apparatus) for at least 10 min before the start of testing to control temperature. Skin surface temperature was recorded immediately before placement in the USV recording chamber via a noncontact HDE Infrared Thermometer to ensure consistent temperatures as lower body temperature of the pup is known to increase USV production (Branchi et al., 1998). Differences in temperature between FLX and VEH pups were not detected, indicating the differences in USV production were not secondary to thermoregulation differences. For recording, pups were individually removed from the home cage and placed into an empty standard mouse cage (28.5 × 17.5 × 12 cm) inside a sound-attenuating chamber (Med Associates). USVs were obtained using an Avisoft UltraSoundGate CM16 microphone, Avisoft UltraSoundGate 416H amplifier, and Avisoft Recorder software (gain = 2 dB, 16 bits, sampling rate = 250 kHz). Pups were recorded for 3 min, after which they were weighed and returned to their home cages inside the warming box. Tissue from a toe was also collected at this time on P5 for genotyping. Frequency sonograms were prepared from recordings in MATLAB [frequency range = 25–120 kHz, FFT (Fast Fourier Transform) size = 512, overlap = 50%, time resolution = 1.024 ms, frequency resolution = 488.2 Hz], and individual syllables and other spectral features were identified and counted from the sonograms according to a previously published method (Dougherty et al., 2013; Rieger and Dougherty, 2016; Maloney et al., 2018), adapted from validated procedures (Holy and Guo, 2005).

#### Developmental reflexes and milestones assessment

Mice were evaluated at several time points for achievement of physical and behavioral milestones of development. A visual check for the presence of detached pinnae was done at P5, and eye opening at P14. Weight was measured at P5, P7, P9, and P14, concurrent with USV recordings and righting reflex testing. To assess surface righting reflex at P14, each mouse was placed in a 50-ml conical tube containing a lid with a hole. When the belly of the mouse was facing down, the conical tube was quickly turned 180° in a smooth motion placing the mouse on its back. The time for the mouse to right itself with all four paws underneath its belly was recorded up to 60 s. Each mouse received three trials, which were averaged for analysis.

#### Juvenile social interaction

Full-contact social behaviors were assessed through juvenile interactions using a procedure adapted from previously published methods (Peñagarikano et al., 2011). Mice were tested between P22–P30 and were paired with an age- and sex-matched C57BL/6J stimulus mouse derived from standard mouse breeding. All mice were weighed before testing. The procedure consisted of three consecutive 10-min trials. During trial 1, the stimulus mouse was habituated to the testing chamber. For trial 2, the test animal was habituated to the chamber while the stimulus mouse was placed in a holding chamber lined with clean corn cob bedding. For the third trial, the stimulus mouse was placed back into the testing chamber with the test mouse and their interactions were recorded for 10 min. The testing chamber was cleaned with 70% ethanol between test animals and the corn cob bedding was replaced. The test apparatus was a transparent enclosure (25 × 15 × 12 cm) containing a layer of clean corn cob bedding on the floor and surrounded by a clear acrylic enclosure measuring 28 × 17.5 × 37.5 cm. A 4-cm diameter hole on the top of the enclosure allowed for placement of a digital video camera (Sony HDR-Cx560V High Definition Handycam camcorder) to record scenes inside the apparatus. The apparatus was housed inside a custom built sound-attenuating chamber (70.5 × 50.5 × 60 cm), which was equipped with two LED infrared lights (Crazy Cart 48-LED CCTV infrared Night Vision Illuminator) to allow for capture of social behaviors in darkness.

Video files in MPG format were acquired in 360 × 240 or 544 × 362 pixel resolution with a frame rate of 25 or 30 frames per second. Videos were minimally post-processed to key only grayscale images, remove associated audio track, and convert to AVI containers before tracking. Simultaneous supervised tracking of both the stimulus and experimental animals was performed in MiceProfiler (de Chaumont et al., 2012) on the Icy platform, with scale value of 0.35 and pixel intensity threshold used to identify mice optimized for each video as necessary to ensure most accurate tracking. This software allows for experimenter supervision of tracking through manual intervention and frame-by-frame correction, and was validated previously by comparing results obtained with MiceProfiler to those obtained by human visual inspection. Social contact data were similar between supervised tracking with MiceProfiler and the experimenter-obtained values (de Chaumont et al., 2012). In the current application, manual corrections of tracking was performed as necessary through the course of each video. Two videos were excluded due to unexpected differences in zoom and resolution, and 11 other videos were excluded, because one mouse left the field of view for a portion of the ten minute testing time.

Tracked videos were then processed using a custom pipeline in MATLAB as follows. MiceProfiler data points for each frame and <x,y> positions of head, center of mass (“body”), and tail were parsed from the XML tracking data, pixel coordinates were converted to centimeters using the real world size of the testing apparatus, and frame number converted to time in seconds using the frame rate. Occasionally isolated frames contained missing data points occur where MiceProfiler does not record a value, and these were recorded as NaN (not-a-number) in MATLAB. Because of these occasional missing values, and jitter which occurs during tracking, data were smoothed using a 11-point moving average smooth, which resulted in more accurate tracking within MiceProfiler. After smoothing, positional values for head, body, and tail were used to estimate two-dimensional kinematics, using the first difference approximation for derivatives: velocity, acceleration, and jerk. Vectors defined by the head and tail positions were used to determine relative orientation of the two mice in the field of view, and final processed data contained the following variables by frame: distance traveled, length of body axis (head-to-tail) and direction (radians) with respect to the field of view (coordinate system <0,0> in lower left), the direction (radians) and magnitude of each 2D component of motion (velocity, acceleration, jerk) for each animal, and inter-animal parameters (angle between both animals and between their velocity vectors, all pairwise distances in cm between head, body, tail), from which total distance traveled and average speed (cm/s) were determined. Thresholds of 3.502 cm for head-to-head distance and 3.125- or 3.145-cm head-to-tail distance were used to define head sniffing and anogenital sniffing behaviors, respectively. These thresholds were determined through examination of the histogram of all head-to-head and head-to-tail distances across all videos and verified by manual inspection of video after applying threshold. After thresholding, bouts of behavior were scored as frames with distances below threshold, and bouts separated by 35 frames or less (≤5.10 or ≤0.17 s) were merged. From these, fraction of total frames for each behavior, as well as number and average duration of bouts of behavior were determined. Measures of overall activity per mouse, such as distance traveled and average speed, were also extracted.

#### Social approach

The social approach task was used to quantify sociability and preference for social novelty, and as previously described (Moy et al., 2004; Dougherty et al., 2013). Sociability was defined here as a tendency to pursue social contact. Preference for social novelty was defined as pursuing social contact with a novel conspecific as compared to a conspecific from a previous interaction. The social approach testing apparatus was a rectangular clear acrylic box divided into three separate chambers each measuring 19.5 × 39 × 22 cm including clear acrylic dividing walls with rectangular openings measuring 5 × 8 cm to allow for movement between chambers, which could be shut off by sliding down clear acrylic doors. This clear acrylic apparatus was housed inside a custom built sound-attenuating chamber (70.5 × 50.5 × 60 cm), lit with LED Flex Ribbon Lights (Commercial Electric, Home Depot) to provide ∼20 lux illumination in the chamber. A small stainless steel conspecific cage (Galaxy Pencil/Utility Cup, Spectrum Diversified Designs, Inc), measuring 10 cm in height and 10 cm in diameter at its base, was placed in each outer chamber, and had vertical bars that allowed minimal contact while preventing fighting. A CCTV camera (SuperCircuits) connected to a PC computer running the software program ANY-maze (Stoelting Co.; RRID: SCR_014289) tracked the movement of the mouse within the apparatus (Dougherty et al., 2013; Miranda et al., 2015) and time spent in each investigation zone surrounding the conspecific cages. The investigation zones encompassed an area of 2 cm around the conspecific cages. Only the head was tracked in the investigation zone to quantify intention to investigate the conspecific. Total distance traveled was also ascertained as an index of general activity levels. The entire apparatus was cleaned between animals with a 2% chlorohexidine diacetate solution (Nolvasan, Zoetis). The conspecific cages were cleaned with 70% ethanol solution between each mouse.

The social approach task consisted of four, consecutive 10-min trials. For the first trial, the mouse was placed in the middle chamber with the doors to the outer chambers shut and allowed 10 min to habituate to the apparatus. During the second trial (habituation trial), the mouse was allowed to freely investigate and habituate to all three chambers for 10 min. Performance of the mouse during the third trial (sociability trial) allowed for the evaluation of sociability to an unfamiliar, sex-matched conspecific (C57BL/6J) placed in one conspecific cage versus an empty conspecific cage. Again, the mouse was allowed to move freely within the apparatus for 10 min. During the fourth trial (preference for social novelty trial), the now familiar conspecific remained in the apparatus, and a new, unfamiliar sex-matched conspecific (C57BL/6J) was placed in the other conspecific cage. The mouse was allowed to move freely within the apparatus for 10 min, and the mouse’s preference for social novelty was quantified. Placement of conspecifics was counterbalanced.

#### Tube test of social dominance

Under laboratory conditions, mice begin to develop social hierarchy behaviors at six weeks of age, which result in dominance ranks within their social groups (Hayashi, 1993). The tube test of social dominance allows for examination of social dominance rank between two pairs of mice after eight weeks of age and was adapted from previously described methods (Wang et al., 2011). The apparatus consisted of a clear acrylic tube measuring 3.6 cm in diameter and 30 cm in length. This task spanned 5 consecutive days. On days 1 and 2, each mouse was exposed to the test apparatus to habituate the animals to the testing tube and to walking through the testing tube to the other side. This was conducted from each side of the tube. On days 3–5, dominance bouts were conducted with sex-matched pairs of FLX and VEH mice, avoiding cage mate pairings. A new pair was used for each bout such that each mouse was paired with three distinct partners, and side of entry was alternated. On each day, male bouts were conducted first, followed by female bouts. For each bout, a small acrylic divider was placed in the center of the tube, prohibiting the animals from crossing the center, and each mouse was allowed to enter the tube from one end. Once the animals met in the center, the divider was lifted and the bout lasted 2 min or until one animal was backed out of the tube by the other (all four paws exiting the tube). The animal remaining in the tube was the winner of the bout (dominant) and the animal that was backed out was the loser of the bout (subordinate). The bouts were recorded with a USB camera connected to a PC laptop (Lenovo) and subsequently scored by an observer. The percentage of bouts won was calculated for each mouse, and compared between groups. The acrylic tube was cleaned with a 2% chlorohexidine diacetate solution (Nolvasan, Zoetis) between each bout.

#### Marble burying task

Marble burying behavior in mice serves as a proxy for repetitive and perseverative digging behavior (Angoa-Pérez et al., 2013), and our procedure was adapted from these previously described methods. The apparatus was a transparent enclosure (47.6 × 25.4 × 20.6 cm) housed within a sound-attenuating chamber (70.5 × 50.5 × 60 cm), lit with LED Flex Ribbon Lights (Commercial Electric, Home Depot) to provide ∼20 lux illumination. Each enclosure was filled with 3 cm of clean, autoclaved corncob bedding. Using a template, 20 clear marbles were placed in five rows of four. For testing, the mouse was placed in the center of the enclosure, and allowed to freely explore for 30 min. The animal was then removed and two independent observers scored buried marbles. A marble was considered buried when at last 2/3 of it was covered by bedding. The average score between the two observers was used for analysis. The correlation between observers’ scores for all marble burying experiments in this study was *r* > .92, *p* = 0.000. In between animals, new fresh, autoclaved bedding was used and all marbles were cleaned thoroughly with 70% ethanol.

#### Spontaneous alternation T-maze

The spontaneous alternation T-maze was used to assess perseverative exploratory behavior and was adapted from previously published methods (Peñagarikano et al., 2011). Testing was conducted under dim overhead lighting. The apparatus was made of opaque acrylic and comprises a 20 × 8.7 cm start chamber with two radiating arms, each measuring 25 × 8.7 cm. Removable doors were used to sequester the animal in the start box, or either maze arm. Testing consisted of 10 consecutive trials, each lasted 2 min or until the animal made an arm choice. For each the first trial, the animal was placed in the start box with the door closed for 2 min to habituate to the apparatus. The door was then removed and the animal allowed to explore either the right or left arm of the maze. An arm choice was determined when the animal entered the arm with all four paws. Then the door to that arm was closed, and the animal allowed to explore it for 5 s. The door was again lifted and the animal was allowed to return to the start box and the door shut. If the animal did not quickly move back to the start area, it was gently guided by placement of a hand or object behind the animal, yet avoiding picking the animal up by the tail and moving back to the start chamber as this can result in a negative association with that arm and impact the spontaneous alternation. After 5 s, the start box door was again lifted to start the next trial. If no arm choice was made after 2 min, the animal was gently guided back to the start box. After 10 consecutive trials, the animal was returned to its home cage and the apparatus cleaned thoroughly with a 2% chlorohexidine diacetate solution (Nolvasan, Zoetis). Each of the two trials was scored as an alternation, a non-alternation or no choice trial. The number of non-alternations and percentage of trials alternating were compared between groups.

#### Tactile sensitivity assessment with von Frey filaments

The tactile sensitivity task assessed reflexive, mechanical sensitivity to a punctate stimulus (von Frey filaments), and was conducted as previously described (Mickle et al., 2015). The testing apparatus consisted of a metal grid surface elevated 63.5 cm, which allowed access to the plantar surface of the animals’ paws. On top set individual acrylic boxes (10 × 10 × 10 cm) open on the bottom and opaque on three sides to prevent visual cues between animals. All mice were acclimated to the testing room 30 min before habituation and testing. On days 1 and 2, all mice were habituated to the testing apparatus for 1 h. On day 3, mice were allowed to acclimate to the testing apparatus for 30 min before start of testing. Eight different von Frey hair filaments (applying 0.04–2 g of force; North Coast Medical and Rehabilitation Products) were applied to the plantar surface of each animal’s hind paw and withdrawal responses were recorded. Presentations started with the lowest filament strength (0.04 g) and increased to the maximum filament strength (2 g). Each filament was applied to the plantar surface of each hind paw five times, and the number of paw withdrawal responses was recorded as percentage of responses. To evaluate the changes in paw withdrawal responses to the whole range of filaments over the testing duration, the area under the curve (AUC) was calculated for each animal.

#### One-hour locomotor activity

A 1-h locomotor activity/exploration test was conducted to assess the general activity, exploratory behavior, and emotionality of the mice. This test also served as a control test to identify any differences in general activity that may interfere with the interpretation of cognitive, social, and/or emotionality tests. The mice were evaluated over a 1-h period in transparent enclosures (47.6 × 25.4 × 20.6 cm). A digital video camera connected to a PC computer running ANY-maze (Stoelting Co.; RRID: SCR_014289) tracked the movement of the animal (Palanisamy et al., 2011; Dougherty et al., 2013) within a 33 × 11-cm central zone and a bordering 5.5-cm peripheral zone. General activity variables (distance traveled and time at rest) along with measures of emotionality, including “time spent,” “distance traveled,” and “entries made into the central zone,” as well as “distance traveled in the peripheral zone” were analyzed. Each enclosure was cleaned with 70% ethanol solution between each mouse.

#### Sensorimotor battery

Balance, strength, and coordination were evaluated by a battery of sensorimotor measures. The battery included walking initiation, ledge, platform, pole, and inclined and inverted screen tests. An observer manually recorded time in hundredths of a second using a stopwatch for each test. Two trials were conducted for each test and the average of the two yielded a single time, which was used in the analyses. To avoid exhaustion effects, the order of the tests during the first set of trials was reversed for the second set of trials. The order of the tests was not counterbalanced between animals so that every animal experienced each test under the same conditions. All tests lasted a maximum of 60 s, except for the pole test, which lasted a maximum of 120 s. The tests are described below.

The walking initiation test assessed the time taken by a mouse to move out of a small area. The mouse was placed on a flat surface inside a square measuring 21 × 21 cm, marked on the surface of a supply cart with white tape. The time for the mouse to leave the square was recorded, i.e., all four limbs concurrently outside of the square. Basic balance ability was assessed by the performance on the ledge and platform tests. The ledge test required the mouse to balance on a clear acrylic ledge, measuring 0.50 cm wide and standing 37.5 cm high. Time the mouse remained on the ledge was recorded. During the platform test, the mouse used basic balance ability to remain on a wooden platform measuring 1.0 cm thick and 3.3 cm in diameter and elevated 27 cm above the floor. The time the mouse was able to balance on the platform was recorded. The pole test was used to evaluate fine motor coordination. The mouse was placed head upward on a vertical pole with a finely textured surface and the time taken by the mouse to turn downward 180° and climb to the bottom of the pole was recorded. The 60°, 90°, and inverted screen tests assessed a combination of coordination and strength. The mouse was placed head oriented downward in the middle of a mesh wire grid measuring 16 squares per 10 cm, elevated 47 cm and inclined to 60° or 90°. The time required by the mouse to turn upward 180° and climb to the top of the screen was recorded. For the inverted screen test, the mouse was placed head oriented downward in the middle of a mesh wire grid measuring 16 squares per 10 cm, elevated 47 cm, and, when it was determined the mouse has a proper grip on the screen, it was inverted to 180°. The time the mouse was able to hold on to the screen without falling off was recorded.

### Experimental design and statistical analysis

All statistical analyses were performed using the IBM SPSS Statistics software (v.24; RRID: SCR_002865) except where otherwise stated. Sample sizes, including litter numbers, for each cohort can be found in Table 1. Before analyses, all data were screened for missing values, fit between distributions and the assumptions of univariate analysis, and homogeneity of variance. ANOVA, including repeated measures (rmANOVA) and mixed model, was used to analyze the behavioral data where appropriate, with main factors of sex and drug exposure. As litter size can influence behavior, and our samples included littermates, we also conducted accompanying analyses of covariance (ANCOVAs) with litter size as the covariate, and report any discrepancies between the results. Linear mixed modeling was used to analyze datasets containing missing values, including spectral or temporal USV features which cannot be assessed if <10 USVs/session are produced. For non-normal distributions, equivalent non-parametric tests were used when available. The Huynh-Feldt adjustment was used to protect against violations of sphericity/compound symmetry assumptions where appropriate. Multiple pairwise comparisons were subjected to Bonferroni correction when appropriate; χ^2^ goodness of fit test was used to assess categorical variables. Tukey’s HSD or the Games–Howell method were used as *post hoc* tests. Probability value for all analyses was *p* < 0.05 except where otherwise stated. Test statistics and other analysis details for each experiment are provided in Tables 2, 4–6, including observed power and effect sizes (Cohen, 1988).

**Table 2.** Statistical summary for Figures 1, 2

Variable		Comparison	Data structure	Statistical test	Output	*p* value	*Post hoc* power	Effect size
Number of USVs	a	*Celf6*-Extended, drug (FLX vs vehicle)	Non-normal	Two-way rmANOVA	*F*_(1,197)_ = 80.854	*p* < 0.000005	1	0.641
	b	*Celf6*-Extended, age × drug × genotype interaction	Non-normal	Two-way rmANOVA	*F*_(3.66,360.87)_ = 2.478	*p* = 0.049	0.667	0.160
	c	Vehicle at P9 Celf6^+/+^ vs Celf6^+/-^ vs Celf6^-/-^	Non-normal	Simple main effect	*F*_(2,591)_ = 15.454	*p* < 0.000005	0.967	0.422
	d	*Celf6* ^+/+^ at P5 FLX vs vehicle	Non-normal	Simple main effect	*F*_(1,591)_ = 5.214	*p* = 0.023	0.625	0.095
	d	*Celf6* ^+/+^ at P7 FLX vs vehicle	Non-normal	Simple main effect	*F*_(1,591)_ = 24.168	*p* < 0.000005	0.998	0.201
	d	*Celf6* ^+/+^ at P9 FLX vs vehicle	Non-normal	Simple main effect	*F*_(1,591)_ = 32.669	*p* < 0.000005	1	0.234
	d	*Celf6* ^+/-^ at P5 FLX vs vehicle	Non-normal	Simple main effect	*F*_(1,591)_ = 8.307	*p* = 0.004	0.821	0.119
	d	*Celf6* ^+/-^ at P7 FLX vs vehicle	Non-normal	Simple main effect	*F*_(1,591)_ = 53.427	*p* < 0.000005	1	0.301
	d	*Celf6* ^+/-^ at P9 FLX vs vehicle	Non-normal	Simple main effect	*F*_(1,591)_ = 35.638	*p* < 0.000005	1	0.246
	d	*Celf6* ^-/-^ at P5 FLX vs vehicle	Non-normal	Simple main effect	*F*_(1,591)_ = 2.724	*p* = 0.099	0.378	0.071
	d	*Celf6* ^-/-^ at P7 FLX vs vehicle	Non-normal	Simple main effect	*F*_(1,591)_ = 24.936	*p* < 0.000005	0.999	0.204
	d	*Celf6* ^-/-^ at P9 FLX vs vehicle	Non-normal	Simple main effect	*F*_(1,591)_ = 1.380	*p* = 0.241	0.217	0.045
	g	Long Prenatal, drug (FLX vs vehicle)	Non-normal	One-way rmANOVA	*F*_(1,43)_ = 18.013	*p* = 0.0001	0.986	0.647
	h	P5 FLX vs vehicle	Non-normal	Simple main effect	*F*_(1,43)_ = 14.689	*p* = 0.0004	0.963	0.585
	h	P7 FLX vs vehicle	Non-normal	Simple main effect	*F*_(1,43)_ = 16.678	*p* = 0.0002	0.979	0.622
	i	P9 FLX vs vehicle	Non-normal	Simple main effect	*F*_(1,43)_ = 3.874	*p* = 0.056	0.486	0.301
	k	Short Prenatal, drug (FLX vs vehicle)	Non-normal	One-way rmANOVA	*F*_(1,43)_ = 0.041	*p* = 0.840	0.052	<0.000
Average duration	e	*Celf6*-Extended, drug (FLX vs vehicle)	Normal	Linear mixed model	*F*_(1,211.820)_ = 31.223	*p* < 0.000005	[0.005, 0.010]	
Simple call pitch range	f	*Celf6*-Extended, drug (FLX vs vehicle)	Normal	Linear mixed model	*F*_(1,170.380)_ = 38.155	*p* < 0.000005	[1895.15, 3675.32]	
	j	Long Prenatal, drug (FLX vs vehicle)	Normal	Linear mixed model	*F*_(1,44.068)_ = 5.256	*p* = 0.027	[251.10, 3901.71]	
Weight	k	*Celf6*-Extended, age (P5 vs P7 vs P9 vs P14)	Normal	Two-way rmANOVA	*F*_(1.46,286.7)_ = 2670.61	*p* < 0.000005	1	3.673
	m	*Celf6*-Extended, drug (FLX vs vehicle)	Normal	Two-way rmANOVA	*F*_(1,197)_ = 56.921	*p* < 0.000005	1	0.537
	n	*Celf6* ^+/+^ at P5 FLX vs vehicle	Normal	Simple main effect	*F*_(1,788)_ = 8.087	*p* = 0.005	0.811	0.101
	n	*Celf6* ^+/+^ at P7 FLX vs vehicle	Normal	Simple main effect	*F*_(1,788)_ = 8.008	*p* = 0.005	0.807	0.101
	n	*Celf6* ^+/+^ at P9 FLX vs vehicle	Normal	Simple main effect	*F*_(1,788)_ = 13.699	*p* = 0.0003	0.959	0.132
	n	*Celf6* ^+/+^ at P14 FLX vs vehicle	Normal	Simple main effect	*F*_(1,788)_ = 34.952	*p* < 0.000005	1	0.209
	n	*Celf6* ^+/-^ at P5 FLX vs vehicle	Normal	Simple main effect	*F*_(1,788)_ = 14.860	*p* = 0.0001	0.971	0.139
	n	*Celf6* ^+/-^ at P7 FLX vs vehicle	Normal	Simple main effect	*F*_(1,788)_ = 18.036	*p* = 0.00002	0.989	0.150
	n	*Celf6* ^+/-^ at P9 FLX vs vehicle	Normal	Simple main effect	*F*_(1,788)_ = 21.454	*p* < 0.000005	0.996	0.167
	n	*Celf6* ^+/-^ at P14 FLX vs vehicle	Normal	Simple main effect	*F*_(1,788)_ = 42.427	*p* < 0.000005	1	0.232
	n	*Celf6* ^-/-^ at P5 FLX vs vehicle	Normal	Simple main effect	*F*_(1,788)_ = 7.462	*p* = 0.006	0.779	0.095
	n	*Celf6* ^-/-^ at P7 FLX vs vehicle	Normal	Simple main effect	*F*_(1,788)_ = 8.869	*p* = 0.003	0.845	0.105
	n	*Celf6* ^-/-^ at P9 FLX vs vehicle	Normal	Simple main effect	*F*_(1,788)_ = 12.822	*p* = 0.0004	0.947	0.128
	n	*Celf6* ^-/-^ at P14 FLX vs vehicle	Normal	Simple main effect	*F*_(1,788)_ = 18.815	*p* = 0.00002	0.991	0.153
	q	*Celf6*-Extended, litter (FLX vs vehicle)	Non-normal	Mann–Whitney *U*	*U*_(203)_ = 4723.5	*p* = 0.301	N/A	0.01
	k	Long Prenatal, age (P5 vs P7 vs P9 vs P14)	Normal	Two-way rmANOVA	*F*_(2.26,97.31)_ = 1231.23	*p* < 0.000005	1	5.330
	m	Long Prenatal, drug (FLX vs vehicle)	Normal	Two-way rmANOVA	*F*_(1,43)_ = 20.887	*p* = 0.00004	0.994	0.697
	n	P5 FLX vs vehicle	Normal	Simple main effect	*F*_(1,172)_ = 4.163	*p* = 0.043	0.528	0.157
	n	P7 FLX vs vehicle	Normal	Simple main effect	*F*_(1,172)_ = 12.029	*p* = 0.0007	0.932	0.264
	n	P9 FLX vs vehicle	Normal	Simple main effect	*F*_(1,172)_ = 27.769	*p* < 0.000005	0.999	0.402
	n	P14 FLX vs vehicle	Normal	Simple main effect	*F*_(1,172)_ = 31.829	*p* < 0.000005	1	0.430
	q	Long Prenatal, litter (FLX vs vehicle)	Non-normal	Mann–Whitney *U*	*U*_(45)_ = 228	*p* = 0.595	N/A	0.01
	k	Short Prenatal, age (P5 vs P7 vs P9 vs P14)	Normal	Two-way rmANOVA	*F*_(1.64,70.58)_ = 892.959	*p* < 0.000005	0.954	4.554
	o	Short Prenatal, drug (FLX vs vehicle)	Normal	Two-way rmANOVA	*F*_(1,43)_ = 25.719	*p* = 0.000008	0.999	0.773
	p	P5 FLX vs vehicle	Normal	Simple main effect	*F*_(1,172)_ = 5.273	*p* = 0.023	0.627	0.176
	p	P7 FLX vs vehicle	Normal	Simple main effect	*F*_(1,172)_ = 13.753	*p* = 0.0003	0.958	0.283
	p	P9 FLX vs vehicle	Normal	Simple main effect	*F*_(1,172)_ = 19.138	*p* = 0.00002	0.992	0.333
	p	P14 FLX vs vehicle	Normal	Simple main effect	*F*_(1,172)_ = 49.019	*p* < 0.000005	1	0.534
	r	Short Prenatal, litter (FLX vs vehicle)	Non-normal	Mann–Whitney *U* test	*U*_(45)_ = 84.5	*p* = 0.00003	N/A	0.35
Latency to righting reflex	s	*Celf6*-Extended, drug (FLX vs vehicle)	Non-normal	Two-way ANOVA	*F*_(1,191)_ = 13.753	*p* = 0.004	0.827	0.212
	t	Long Prenatal, drug (FLX vs vehicle)	Non-normal	Mann–Whitney *U*	*U*_(45)_ = 223.0	*p* = 0.545	N/A	0.01
	u	Short Prenatal, drug (FLX vs vehicle)	Non-normal	Mann–Whitney *U*	*U*_(45)_ = 187.5	*p* = 0.140	N/A	0.05

Effect size for *F* tests reported as Cohen’s *f* (Cohen, 1988; interpretation: 0.01 = small; 0.25 = medium; 0.40 = large) and for nonparametric tests reported as η^2^. 95% confidence intervals reported for linear mixed models.

## Results

### Development of SSRI maternal exposure models

To determine the potential of maternal SSRI exposure to induce behavioral disruptions in offspring reminiscent of ASD symptomatology, we exposed mouse dams to FLX during gestation and lactation and examined offspring behaviors during development, the juvenile stage, and adulthood (Fig. 1*A*; Table 1). We included both C57BL/6J line and the *Celf6* mutant line to examine the influence of FLX exposure alone or in combination with a genetically vulnerable background. We also examined different pre- and postnatal durations of FLX to establish periods of vulnerability. Epidemiological studies are inconsistent regarding the trimesters of pregnancy most vulnerable to SSRI-induced ASD risk. To address this, we used three FLX durations, corresponding to periods of brain development approximating the trimesters of human pregnancy. Our designation of “Extended FLX” corresponded to the entire duration of the pregnancy and a recommended period of nursing (one year) in humans (E0–P14; Dobbing and Sands, 1979; Levitt, 2003). Both *Celf6* and C57BL/6J mice were exposed for this duration (*Celf6*-Extended and C57-Extended). “Long Prenatal” (E0–P0) exposure approximated the first and second trimesters of human pregnancy. “Short Prenatal” (E0–E16) approximated the first trimester of human pregnancy (Fig. 1*A*). Only C57BL/6J mice were used for prenatal-only exposures. Overall, our experimental design enabled analysis of both gene × environment interaction and exposure duration effects on behaviors relevant to the symptoms of ASD.

### Maternal FLX disrupts early communicative behavior in pup offspring

We examined developmental behavior, physical milestones and reflexes in our FLX mice. Quantification of USV production and features served to assess neurodevelopmental progress as well as to examine early affective and communicative behavior known to influence maternal care behavior (Haack et al., 1983). At P5, P7, and P9, we observed robust decreases in USVs when FLX lasted through pregnancy. No influence of sex was observed for developmental analyses, therefore all data reported below are collapsed for sex. Output from statistical tests is fully reported in Table 2. Specifically, *Celf6*-Extended exposure to FLX reduced USVs independent of *Celf6* genotype (*p* < 0.000005^a^; Fig. 1*B*), yet an interaction with genotype was also observed (*p* = 0.049^b^). *Celf6* mutation reduced USVs in VEH-exposed pups (*p* < 0.000005^cb^), replicating previous work (Dougherty et al., 2013). Further *post hoc* tests revealed FLX-induced USV reduction at each age across all mice (*p* < 0.024^d^), except for P5 and P9 *Celf6* ^-/-^ mice when USVs were already low due to mutation. Robust reductions in the duration time of calls (*p* < 0.000005^e^; Fig. 1*C*) and the pitch range of simple calls pups (*p* < 0.000005^f^; Fig. 1*D*) were observed in FLX. *Celf6* mutation did not influence spectral or temporal features of USVs alone or through an interaction with extended FLX .

Since the impact of FLX alone was so strong, and independent of *Celf6* mutation in the *Celf6*-Extended cohort, we examined the impact of prenatal-only exposure to FLX on USV in C57BL/6J mice. Long Prenatal exposure to FLX also reduced USVs (*p* = 0.0001^g^; Fig. 1*E*). This FLX-induced reduction occurred at P5 and P7 (*p* < 0.0005^h^), with a trend at P9 (*p* = 0.056^i^). Examination of spectral and temporal features showed Long Prenatal exposure only altered the pitch range of simple calls (*p* = 0.027^j^; Fig. 1*F*). Short Prenatal exposure to FLX did not influence pup USV production (*p* = 0.840^k^; Fig. 1*G*). Taken together, these findings suggest FLX, when continued through pregnancy, induced early communicative deficits in mice in the form of USV reductions, yet FLX limited to early pregnancy did not influence production rate. Further, the effect of FLX on USVs was so robust that we did not have the ability to observe additional interaction with *Celf6* mutation.

### Developmental assessment of physical milestones and reflexes

**Figure 2.**
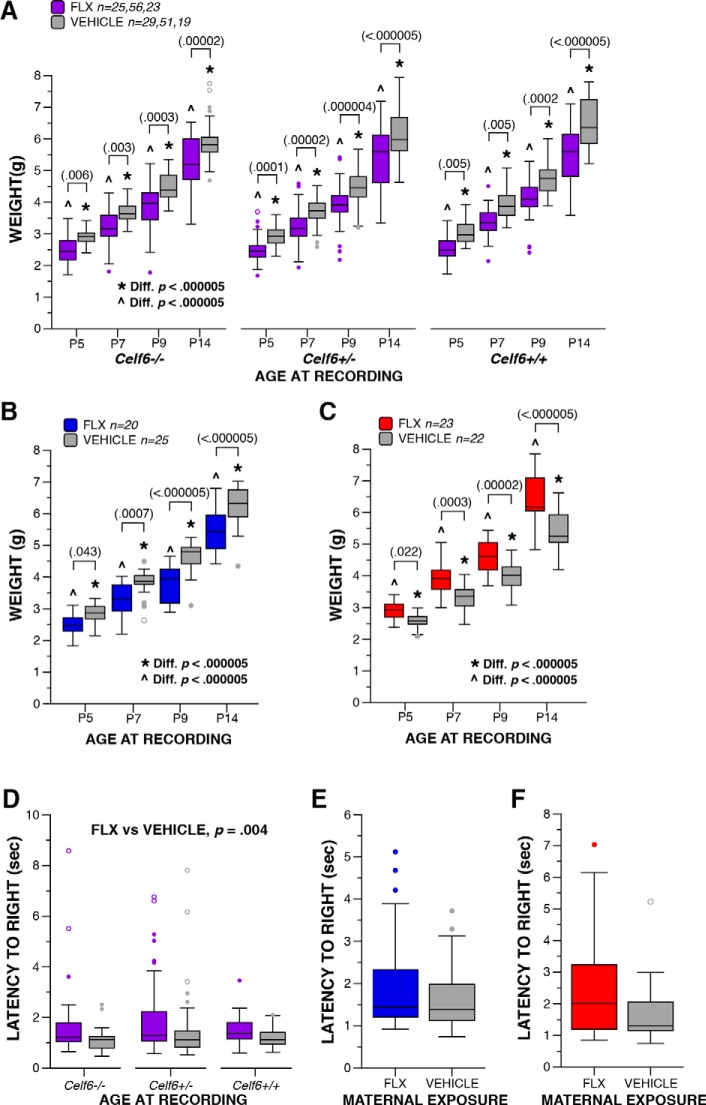
Maternal FLX exposure decreases weight reduction and alters righting reflex pups. ***A–C***, Boxplot of weight at P5, P7, P9, and P14 of *Celf6*-Extended (***A***; drug, *p* < 0.000005), Long Prenatal (***B***; drug, *p* = 0.00004), and Short Prenatal (***C***; drug, *p* = 0.000008) FLX and VEH pups. All mice gained weight with age. ***D–F***, Boxplot of the latency to exhibit a righting reflex at P14 by *Celf6*-Extended (***E***; drug, *p* = 0.004), Long Prenatal (***F***; drug, *p* = 0.545), and Short Prenatal (***G***; drug, *p* = 0.140) FLX and VEH pups; * denotes significant difference across ages at *p* < 0.000005 within VEH-exposed mice; ^ denotes significant difference across ages at *p* < 0.000005 within FLX-exposed mice. For boxplots, thick horizontal lines signify respective group medians, boxes are 25th–75th percentiles, whiskers are 1.5 × IQR, closed and open circles depict outliers.

USV suppression may be a consequence of perturbation of specific CNS circuits due to FLX exposure. However, an alternative explanation is that USV is suppressed by a FLX-induced gross developmental delay. To explore this possibility, we examined other developmental traits of FLX pups. As a measure of general health, we compared the weight of FLX and VEH mice on P5, P7, P9, and P14. Mice in all cohorts increased in weight across developmental time points, as expected (*p* < 0.000005^l^; Fig. 2*A–C*), yet the duration of FLX exposure influenced weight. All *Celf6*-Extended and Long Prenatal FLX mice weighed less than VEH pups (*p* < 0.00005^m^; Fig. 2*A*,*B*) regardless of genotype at each age examined (*p* < 0.044^n^). Interestingly, Short Prenatal FLX resulted in increased weight compared to VEH (*p* = 0.000008^°^; Fig. 2*C*) at all ages examined (*p* < 0.023^p^). However, these weight differences are less likely a result of the E0–E16 FLX exposure and more likely an indirect result due to decreased litter size in this cohort. Analysis of litter sizes across treatment groups in each cohort revealed no effect of litter size for the *Celf6*-Extended and Long Prenatal groups (*p* = 0.3^q^ and *p* = 0.582^q^, respectively; Table 2), indicating the weight differences are due to the FLX treatment, and replicating previous findings (Svirsky et al., 2016). However, a significant difference in litter sizes between the FLX- and VEH-exposed Short Prenatal groups was observed (*p* = 0.000006^r^; FLX, *M* = 5.65, *SD* = 1.15; VEH, *M* = 7.55, *SD* = 1.30), indicating the increase in weight in the FLX mice is likely a result of their smaller average litter sizes. The addition of litter size as a covariate in the model did not change the overall results of weight analyses for the three cohorts. However, the influence of drug on weights only at P5 for the Long and Short Prenatal animals was found to be marginally significant (*p* = 0.059) and non-significant (*p* = 0.304) in the ANCOVA model. Further assessment of developmental milestones revealed that FLX exposure had no effect on the timing of pinna detachment (by P5) or eye opening (by P14; data not shown). To assess early gross locomotor abilities and to evaluate general body strength, we examined righting reflex at P14. When collapsed across genotypes, FLX pups in the *Celf6*-Extended cohort exhibited a longer latency to right compared to VEH pups (*p* = 0.004^s^; Fig. 2*D*). No difference in latency to right was observed in the Long Prenatal cohort (*p* = 0.537^t^; Fig. 2*E*), or in the Short Prenatal cohort (*p* = 0.137^u^; Fig. 2*F*). The developmental data show age-appropriate physical milestones were achieved, indicating FLX did not induce robust developmental delay; however, developmental reflexes were minimally influenced by FLX and weight was affected across development suggesting FLX exposure did induce some developmental perturbation in pups. Thus, the reduction in USVs cannot be completely decoupled from FLX influence on developmental progression.

To confirm the presence of FLX and its active metabolite NFLX in the pup brains, we examined levels of these compounds in whole brain tissue of P9 pup receiving Extended drug exposure, as well as in the whole brain tissue from dams to compare pups levels to that of direct drug exposure.

Given the half-life of FLX (∼6 h^1^) and its active metabolite NFLX (∼15 h^2^) *in vivo*, both should be well cleared by the time the juvenile and adult offspring were analyzed. However, we shared the reviewers interest in whether the early postnatal time points might be influenced by ongoing FLX/NFLX in the brain.

To confirm the drug was reaching the developing brain, HPLC was used to measure levels of FLX and its active metabolite NFLX in whole brains of pups exposed to extended maternal FLX exposure. We found FLX and NFLX were both present in the P9 pup brain during maternal FLX exposure, and neither present in the VEH-exposed control brains (Table 3). The levels of FLX and NFLX in the pups were ∼43% and 32%, respectively, of that measured in an equal amount of dam brain tissue. These data indicate that FLX and NFLX are active in the offspring brain during maternal exposure, suggesting the 5-HT system is targeted at this time. Given the half-life of FLX (∼6 h) and its active metabolite NFLX (∼15 h) *in vivo*, both should be well-cleared by juvenile and adult ages (Holladay et al., 1998; Marken and Munro, 2000). Thus while the alterations in USV behavior might be impacted by the acute levels of FLX and NFLX, the later behavioral alterations must reflect long-term consequences of transient exposure.

**Table 3.** Brain levels of FLX and NFLX (μg/g) from extended exposure dams and P9 pups

	FLX		NFLX	
	M	SD	M	SD
Dam FLX	4534.5	1540.8	6122.5	2003.6
Dam VEH	<LOD	<LOD	<LOD	<LOD
Pup FLX	1962.3	3398.9	1957.0	943.8
Pup VEH	<LOD	<LOD	<LOD	<LOD

Limit of detection (LOD) was 164 ng/g for FLX and 320 ng/g for NFLX.

### Maternal FLX disrupts adult social behaviors

Deficits in social communication and social interaction are varied among autistic individuals, and include failure to initiate or respond to social interaction, abnormal social approach, and difficulties adjusting behavior to suit various social contexts (American Psychiatric Association, 2013). Therefore, we tested our mice in multiple social behavior assays, each designed to assess a distinct aspect of social behavior. The full-contact juvenile interaction assay was used to assess social interaction behaviors in FLX mice, and in adulthood, we examined social approach behaviors and possible disruptions to behaviors in the specific context of social dominance hierarchies.

**Figure 3.**
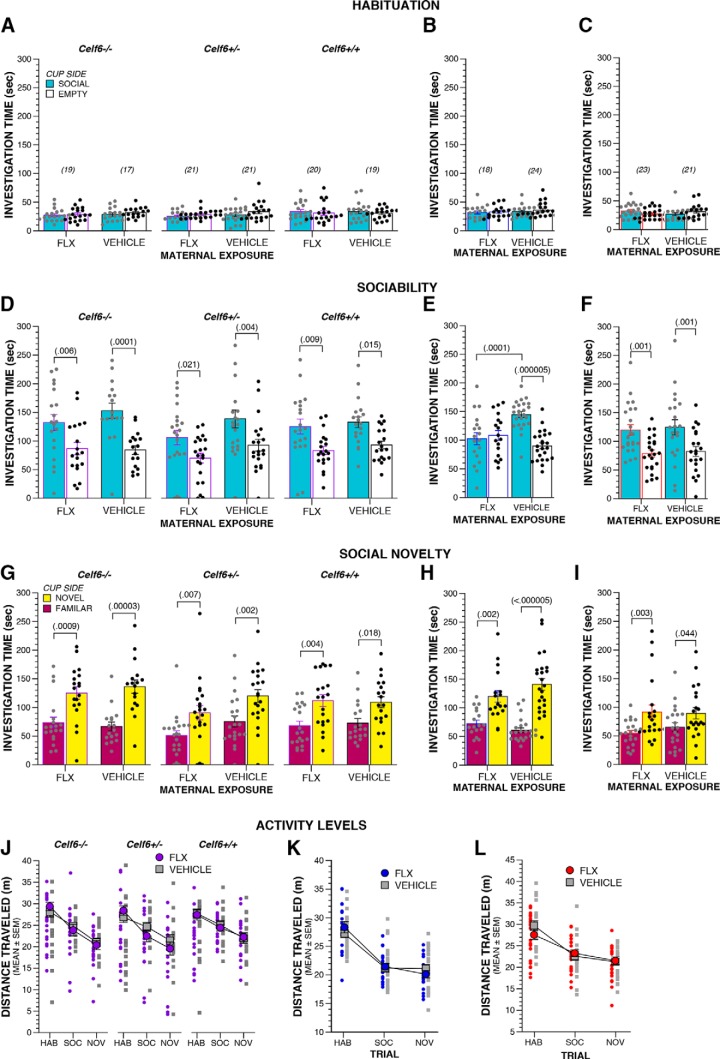
Adult sociability is disrupted by maternal FLX exposure only during pregnancy. ***A–C***, Time spent investigating social and empty cup zones during the social approach habituation trial by *Celf6*-Extended (***A***), Long Prenatal (***B***), and Short Prenatal (***C***) FLX and VEH mice. ***D*–*F***, Time spent investigating social and empty cups during the sociability trial of the social approach test by *Celf6*-Extended (***D***; drug, *p* = 0.020), Long Prenatal (***E***; stimulus × drug, *p* = 0.0004), and Short Prenatal (***F***; stimulus × drug, *p* = 0.962) FLX and VEH mice. ***G–I***, Boxplots of time spent investigating cups containing novel or familiar conspecifics during the preference for social novelty trial of the social approach test *Celf6*-Extended (***G***; stimulus, *p* < 0.000005), Long Prenatal (***H***; stimulus, *p* < 0.000005), and Short Prenatal (***I***; stimulus, *p* = 0.001) FLX and VEH mice. ***J–L***, Distance traveled during the social approach task by *Celf6*-Extended (***J***), Long Prenatal (***K***), and Short Prenatal (***L***) FLX and VEH mice. Data are mean ± SEM, with individual data points represented as filled circles/squares (***A–I***: social/familiar zone, gray; empty/novel zone, black; ***J–L***: FLX, purple/blue/red; WT, gray).

Maternal FLX exposure disrupted social approach and specific social hierarchy behaviors in adulthood, but not juvenile social interactions. Significant interactions between sex and drug exposure were not observed, therefore results are reported collapsed across sex. Output from statistical tests is fully reported in Table 4. During the social approach habituation trial, no side bias was observed for any cohort (Fig. 3*A–C*). In the *Celf6*-Extended exposure group, when collapsed for genotype, VEH mice spent more time compared to FLX mice investigating both stimuli overall (*p* = 0.020^v^; Fig. 3*D*), and more time investigating the social stimulus (*p* = 0.028^w^). Yet, the expected preference for social stimulus was observed for all FLX and VEH *Celf6* mutant and WT mice (*p* < 0.022^x^). As *Celf6* mutation did not potentiate the impact of FLX on sociability behavior, we continued our examination of social approach behaviors without manipulation of *Celf6* genotype for the Long and Short Prenatal cohorts. Long Prenatal exposure resulted in disruptions to sociability (*p* = 0.0004^y^). FLX mice failed to display a preference for the social stimulus (*p* = 0.645^z^; VEH, *p* < 0.000005^aa^; Fig. 3*E*), and spent significantly less time investigating the social stimulus compared to VEH mice (*p* = 0.0001^bb^). Short Prenatal exposure did not disrupt sociability (*p* = 0.962^cc^): both FLX and VEH spent more time investigating the social stimulus than the empty cup (FLX, *p* = 0.001^dd^; VEH, *p* = 0.001^ee^; Fig. 3*F*), and a similar time was spent investigating the social stimulus by both groups (*p* = 0.726^ff^). Finally, during the preference for social novelty trial, again the *Celf6*-Extended cohort VEH mice showed a strong trend for investigating the objects more overall compared to FLX mice (*p* = 0.065^gg^), when collapsed for genotype. For all cohorts, more time was spent investigating the novel mouse compared to the familiar mouse in all cohorts (*p* < 0.045^hh^; Fig. 3*G–I*). Comparable activity levels were detected for all groups in this task (Fig. 3*J–L*), ruling out hypoactivity as a confound. Taken together, these data indicate maternal FLX influenced sociability only when continued throughout pregnancy. We did not demonstrate a strong impact of FLX exposure limited to early pregnancy or extended into postnatal development on adult sociability in our mice.

**Table 4.** Statistical summary for Figures 3, 4

Variable		Comparison	Data structure	Statistical test	Output	*p* value	*Post hoc* power	Effect size
Sociability investigation time	v	*Celf6*-Extended, drug (FLX vs vehicle)	Normal	Two-way rmANOVA	*F*_(1,111)_ = 5.608	*p* = 0.020	0.651	0.225
	x	*Celf6* ^++^ FLX social vs empty stimulus	Normal	Simple main effect	*F*_(1,111)_ = 6.983	*p* = 0.009	0.745	0.250
	x	*Celf6* ^+/-^ FLX social vs empty stimulus	Normal	Simple main effect	*F*_(1,111)_ = 5.440	*p* = 0.021	0.638	0.222
	x	*Celf6* ^-/-^ FLX social vs empty stimulus	Normal	Simple main effect	*F*_(1,111)_ = 7.821	*p* = 0.006	0.792	0.266
	x	*Celf6*^++^ vehicle social vs empty stimulus	Normal	Simple main effect	*F*_(1,111)_ = 5.998	*p* = 0.016	0.680	0.232
	x	*Celf6* ^+/-^ vehicle social vs empty stimulus	Normal	Simple main effect	*F*_(1,111)_ = 8.852	*p* = 0.004	0.839	0.283
	x	*Celf6*^-/-^ vehicle social vs empty stimulus	Normal	Simple main effect	*F*_(1,111)_ = 15.898	*p* = 0.0001	0.977	0.378
	w	Social stimulus FLX vs vehicle	Normal	Simple main effect	*F*_(1,222)_ = 4.895	*p* = 0.028	0.596	0.150
	y	Long Prenatal, stimulus × drug interaction	Normal	One-way rmANOVA	*F*_(1,40)_ = 14.627	*p* = 0.0004	0.962	0.605
	z	FLX social vs empty stimulus	Normal	Simple main effect	*F*_(1,40)_ = 0.216	*p* = 0.645	0.074	0.071
	aa	Vehicle social vs empty stimulus	Normal	Simple main effect	*F*_(1,40)_ = 28.149	*p* < 0.000005	0.999	0.839
	bb	Social stimulus FLX vs vehicle	Normal	Simple main effect	*F*_(1,80)_ = 16.659	*p* = 0.0001	0.981	0.456
	cc	Short Prenatal, stimulus × drug interaction	Normal	One-way rmANOVA	*F*_(1,42)_ = 0.002	*p* = 0.962	0.050	0.007
	dd	FLX social vs empty stimulus	Normal	Simple main effect	*F*_(1,42)_ = 12.337	*p* = 0.001	0.929	0.032
	ee	Vehicle social vs empty stimulus	Normal	Simple main effect	*F*_(1,42)_ = 11.715	*p* = 0.001	0.917	0.032
	ff	Social stimulus FLX vs vehicle	Normal	Simple main effect	*F*_(1,84)_ = 0.124	*p* = 0.726	0.064	0.032
Social novelty investigation time	gg	*Celf6*-Extended, drug (FLX vs vehicle)	Normal	Two-way rmANOVA	*F*_(1,111)_ = 3.468	*p* = 0.065	0.455	0.176
	hh	*Celf6* ^++^ FLX Fam vs novel stimulus	Normal	Simple main effect	*F*_(1,111)_ = 8.845	*p* = 0.004	0.838	0.283
	hh	*Celf6* ^+/-^ FLX Fam vs novel stimulus	Normal	Simple main effect	*F*_(1,111)_ = 7.618	*p* = 0.007	0.781	0.261
	hh	*Celf6* ^-/-^ FLX Fam vs novel stimulus	Normal	Simple main effect	*F*_(1,111)_ = 11.659	*p* = 0.0009	0.923	0.324
	hh	*Celf6* ^++^ vehicle Fam vs novel stimulus	Normal	Simple main effect	*F*_(1,111)_ = 5.812	*p* = 0.018	0.666	0.229
	hh	*Celf6* ^+/-^ vehicle Fam vs novel stimulus	Normal	Simple main effect	*F*_(1,111)_ = 9.616	*p* = 0.002	0.867	0.295
	hh	*Celf6* ^-/-^ vehicle Fam vs novel stimulus	Normal	Simple main effect	*F*_(1,111)_ = 18.954	*p* = 0.00003	0.991	0.413
	hh	Long Prenatal, stimulus (Fam vs novel cup)	Non-normal	One-way rmANOVA	*F*_(1,40)_ = 46.742	*p* < 0.000005	1	1.081
	hh	FLX Fam vs novel stimulus	Non-normal	Simple main effect	*F*_(1,40)_ = 11.365	*p* = 0.002	0.908	0.533
	hh	Vehicle Fam vs novel stimulus	Non-normal	Simple main effect	*F*_(1,40)_ = 42.911	*p* < 0.000005	1	1.037
	hh	Short Prenatal, stimulus (Fam vs novel cup)	Non-normal	One-way rmANOVA	*F*_(1,40)_ = 13.815	*p* = 0.001	0.952	0.588
	hh	FLX Fam vs novel stimulus	Non-normal	Simple main effect	*F*_(1,40)_ = 10.119	*p* = 0.003	0.874	0.503
	hh	Vehicle Fam vs novel stimulus	normal	Simple main effect	*F*_(1,40)_ = 4.307	*p* = 0.044	0.526	0.328
Percent tube test bouts won	ii	C57-Extended FLX, compared to 50%	Non-normal	One-spl Wilcoxon	*Z* = 2.418	*p* = 0.016	N/A	0.24
	jj	C57-Extended vehicle, compared to 50%	Non-normal	One-spl Wilcoxon	*Z* = -2.398	*p* = 0.016	N/A	0.24
	kk	Long Prenatal FLX, compared to 50%	Non-normal	One-spl Wilcoxon	*Z* = -2.356	*p* = 0.018	N/A	0.69
	kk	Long Prenatal vehicle, compared to 50%	Non-normal	One-spl Wilcoxon	*Z* = 1.873	*p* = 0.061	N/A	0.44
	ll	Short Prenatal FLX, compared to 50%	Non-normal	One-spl Wilcoxon	*Z* = -1.907	*p* = 0.057	N/A	0.20
	ll	Short Prenatal vehicle, compared to 50%	Non-normal	One-spl Wilcoxon	*Z* = 1.691	*p* = 0.091	N/A	0.16
Adult weight	mm	C57-Extended, drug (FLX vs vehicle)	Normal	Two-way ANOVA	*F*_(1,12)_ = 0.475	*p* = 0.504	0.097	0.199
	nn	Long Prenatal, drug (FLX vs vehicle)	Normal	Two-way ANOVA	*F*_(1,40)_ = 8.096	*p* = 0.007	0.793	0.449
	oo	Short Prenatal, drug (FLX vs vehicle)	Normal	Two-way ANOVA	*F*_(1,40)_ = 1.796	*p* = 0.188	0.258	0.212

Effect sample size for *F* tests reported as Cohen’s *f* (Cohen, 1988; interpretation: 0.01 = small; 0.25 = medium; 0.40 = large) and for nonparametric tests reported as η^2^.

As *Celf6* genotype did not influence sociability in the social approach task, we chose to examine full-contact social behaviors in C57BL/6J juveniles in a separate C57-Extended cohort. We did not observe abnormal social interactions in these mice in the juvenile interaction assay. Specifically, FLX and VEH mice exhibited a comparable number and duration of anogenital and head-to-head sniffing, and sniffing behaviors directed toward FLX and VEH mice by the stimulus partners were also similar (data not shown). Unlike the social approach task, we did not observe altered social behaviors in the juvenile interaction assay. However, in social approach only the FLX mouse has control over timing and duration of interactions, while in juvenile interaction, deficits in social behaviors with FLX treatment could be masked because interactions were also initiated by the unexposed stimulus mouse.

**Figure 4.**
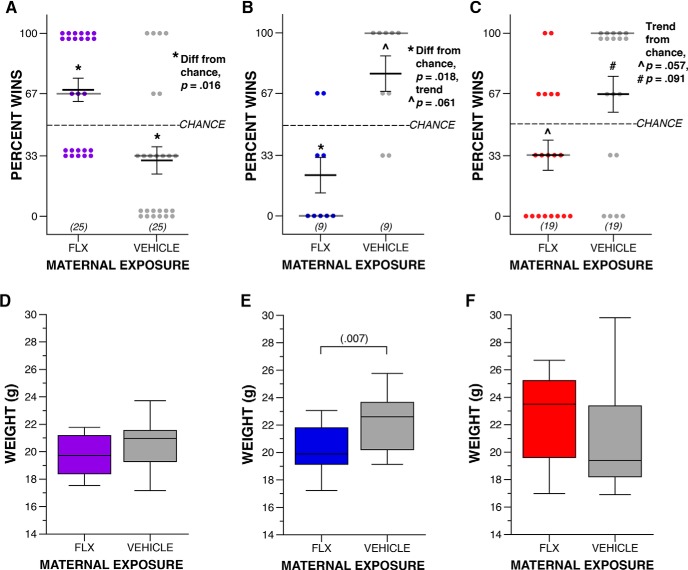
Maternal FLX disrupts adult social dominance behaviors. ***A–C***, Dot plots of percentage of wins during tube test of social dominance between FLX and VEH adult mice in the C57-Extended (***A***; * denotes significant difference from chance at *p* = 0.016), Long Prenatal (***B***; * denotes significant difference from chance at *p* = 0.018; ^ denotes marginally significant difference from chance at *p* = 0.061), and Short Prenatal cohorts (***C***; **^** denotes marginally significant difference from chance at *p* = 0.057; # denotes marginally significant difference from chance at *p* = 0.091). Crosshairs represent mean ± SEM, and dark gray lines represent medians. ***D–F***, Boxplots of weight of C57-Extended (***D***; drug, *p* > 0.05), Long Prenatal (***E***; drug, *p* = 0.007), and Short Prenatal (***F***; drug, *p* > 0.05) FLX and VEH adult mice. For boxplots, thick horizontal lines signify respective group medians, boxes are 25th–75th percentiles, whiskers are 1.5 × IQR, closed and open circles depict outliers.

Finally, we examined social hierarchy behaviors in our mice to determine whether maternal FLX exposure influences behavior in this specific social context. Groups of mice display social hierarchies with dominant and submissive group members (Hayashi, 1993), and we assessed this using the tube test for social dominance. For this task, sex-matched mice from different experimental groups are directly compared. Due to the complexity of experimental groups in the *Celf6*-Extended cohort, we only examined tube test behavior between FLX and VEH mice in the C57-Extended cohort. We observed an interesting influence of FLX duration on dominance. C57-Extended FLX resulted in increased dominant behavior (Fig. 4*A*, FLX wins greater than by chance, *p* = 0.016^ii^; VEH wins fewer than by chance, *p* = 0.016^jj^). In contrast, both maternal FLX cohorts restricted to prenatal development induced submissive behaviors in adulthood: Long Prenatal FLX resulted in fewer wins relative to chance (*p* = 0.018^kk^; Fig. 4*B*). Short Prenatal exposure influenced dominance behavior less strongly, resulting in fewer bouts won than expected by chance by FLX-exposed mice, which did not reach statistical significance (*p* = 0.057^ll^; Fig. 4*C*). These alterations in dominance were not due to differences in animal size between drug exposure groups as adult weights did not correspond to increased dominance in a simple way. Specifically, at available power we did not detect differences in adult weight in C57-Extended FLX mice (*p* = 0.504^mm^; Fig. 4*D*). Long Prenatal FLX resulted in a decrease in weight compared to VEH, that was independent of sex (*p* = 0.007^nn^; Fig. 4*E*) and dominance performance. We also did not detect a weight difference among mice of the Short Prenatal cohort (*p* = 0.188^oo^; Fig. 4*F*). Taken together, these data suggest perinatal FLX exposure via the mother influences social behaviors during adulthood, long after drug exposure occurred, with specific disruptions to sociability and behavior in the specific context of dominance. Further, prenatal versus postnatal exposure may differentially influence behavioral circuits underlying dominance behaviors.

### Extended maternal FLX induces repetitive, restricted patterns of behavior

Similar to our analysis of social behaviors, we assessed a range of rodent tasks relevant to repetitive and restricted patterns of behavior to fully characterize the influence of FLX on this symptom domain. In humans, these symptoms can manifest: as stereotyped or repetitive motor movements, use of objects, or speech; insistence on sameness, inflexible adherence to routines or patterns; or highly restricted interests. This domain also includes hyper- or hypo-reactivity to sensory input (American Psychiatric Association, 2013). In our mice, we used the marble burying task to examine compulsive digging, spontaneous alternation T-maze to test inflexible adherence to behavior patterns (perseveration), and von Frey filaments to gauge reactivity to tactile stimulation. Output from statistical tests for this section is fully reported in Table 5.

**Table 5.** Statistical summary for Figure 5

Variable		Comparison	Data structure	Statistical test	Output	*p* value	*Post hoc* power	Effect size
Marbles buried	pp	*Celf6*-Extended, genotype (Celf6^+/+^ vs Celf6^+/-^ vs Celf)	Normal	Two-way ANOVA	*F*_(2,117)_ = 6.209	*p* = 0.03	0.886	0.326
	qq	*Celf6*-Extended, drug × genotype interaction	Normal	Two-way ANOVA	*F*_(2,117)_ = 3.559	*p* = 0.032	0.651	0.246
	rr	*Celf6* ^+/+^ FLX vs vehicle	Normal	Simple main effect	*F*_(1,117)_ = 14.687	*p* = 0.0002	0.967	0.355
	ss	C57-Extended, drug (FLX vs vehicle)	Normal	One-way ANOVA	*F*_(1,63)_ = 1.080	*p* = 0.303	0.176	0.132
	tt	Long Prenatal, drug (FLX vs vehicle)	Normal	One-way ANOVA	*F*_(1,42)_ = 1.456	*p* = 0.234	0.218	0.188
	tt	Short Prenatal, drug (FLX vs vehicle)	Normal	One-way ANOVA	*F*_(1,42)_ = 0.168	*p* = 0.684	0.069	0.063
Percent alternating trials	ww	*Celf6*-Extended, drug (FLX vs vehicle)	Non-normal	Two-way ANOVA	*F*_(1,117)_ = 16.205	*p* = 0.0001	0.979	0.373
	ww	*Celf6* ^+/+^ FLX vs vehicle	Non-normal	Simple main effect	*F*_(1,117)_ = 6.857	*p* = 0.010	0.738	0.241
	ww	*Celf6* ^+/-^ FLX vs vehicle	Non-normal	Simple main effect	*F*_(1,117)_ = 10.292	*p* = 0.002	0.889	0.297
	uu	*Celf6*-Extended vehicle *Celf6* ^+/+^, compared to 50%	Non-normal	One-spl Wilcoxon	*Z* = 3.231	*p* = 0.001	N/A	0.55
	uu	*Celf6*-Extended vehicle *Celf6* ^+/-^, compared to 50%	Non-normal	One-spl Wilcoxon	*Z* = 4.228	*p* = 0.00002	N/A	0.81
	uu	*Celf6*-Extended vehicle *Celf6* ^-/-^, compared to 50%	Non-normal	One-spl Wilcoxon	*Z* = 3.470	*p* = 0.0005	N/A	0.71
	xx	C57-Extended, drug (FLX vs vehicle)	Non-normal	Mann–Whitney *U*	*U*_(31)_ = 67.5	*p* = 0.032	N/A	0.15
	vv	C57-Extended, vehicle compared to 50%	Non-normal	One-spl Wilcoxon	*Z* = 2.958	*p* = 0.003	N/A	0.55
	vv	C57-Extended, FLX compared to 50%	Non-normal	One-spl Wilcoxon	*Z* = 0.608	*p* = 0.543	N/A	0.03
	aaa	Long Prenatal, drug (FLX vs vehicle)	Non-normal	Mann–Whitney *U*	*U*_(44)_ = 221.5	*p* = 0.706	N/A	<0.01
	eee	Long Prenatal, vehicle compared to 50%	Non-normal	One-spl Wilcoxon	*Z* = 2.303	*p* = 0.021	N/A	0.22
	fff	Long Prenatal, FLX compared to 50%	Non-normal	One-spl Wilcoxon	*Z* = 1.608	*p* = 0.108	N/A	0.14
	bbb	Short Prenatal, drug (FLX vs vehicle)	Normal	One-way ANOVA	*F*_(1,40)_ = 1.555	*p* = 0.220	0.229	0.196
	ggg	Short Prenatal, vehicle compared to 50%	Normal	One-sample t-test	*t*_(19)_ = 3.324	*p* = 0.004	0.883	0.743
	ggg	Short Prenatal, FLX compared to 50%	Normal	One-sample t-test	*t*_(21)_ = 2.541	*p* = 0.019	0.679	0.542
No. of non-alternation trials	yy	*Celf6*-Extended, drug (FLX vs vehicle)	Non-normal	Two-way ANOVA	*F*_(1,117)_ = 16.290	*p* = 0.0001	0.979	0.373
	yy	*Celf6* ^+/+^ FLX vs vehicle	Non-normal	Simple main effect	*F*_(1,117)_ = 6.893	*p* = 0.010	0.740	0.244
	yy	*Celf6* ^+/-^ FLX vs vehicle	Non-normal	Simple main effect	*F*_(1,117)_ = 9.267	*p* = 0.003	0.855	0.281
	zz	C57-Extended, drug (FLX vs vehicle)	Non-normal	Mann–Whitney *U*	*U*_(31)_ = 72	*p* = 0.054	N/A	0.13
	ccc	Long Prenatal, drug (FLX vs vehicle)	Non-normal	Mann–Whitney *U*	*U*_(44)_ = 171.5	*p* = 0.214	N/A	0.04
	ddd	Short Prenatal, drug (FLX vs vehicle)	Normal	One-way ANOVA	*F*_(1,40)_ = 1.555	*p* = 0.220	0.229	0.196
Percent response trials	hhh	C57-Extended, drug × filament interaction	Non-normal	One-way rmANOVA	*F*_(7,98)_ = 3.113	*p* = 0.005	0.932	0.472
	iii	0.16 g filament FLX vs vehicle	Normal	Simple main effect	*F*_(1,112)_ = 4.104	*p* = 0.045	0.519	0.19
	iii	0.4 g filament FLX vs vehicle	Normal	Simple main effect	*F*_(1,112)_ = 13.053	*p* = 0.0005	0.948	0.34
	iii	0.6 g filament FLX vs vehicle	Normal	Simple main effect	*F*_(1,112)_ = 13.357	*p* = 0.0004	0.952	0.35
	jjj	C57-Extended AUC, drug (FLX vs vehicle)	Non-normal	Mann–Whitney *U*	*U*_(16)_ = 15.5	*p* = 0.096	N/A	0.19

Effect size for *F* tests reported as Cohen’s *f* (Cohen, 1988; interpretation: 0.01 = small; 0.25 = medium; 0.40 = large), for *t* tests as Cohen’s *d* (interpretation: 0.2 = small; 0.5 = medium; 0.8 = large) and for nonparametric tests reported as η^2^.

**Figure 5.**
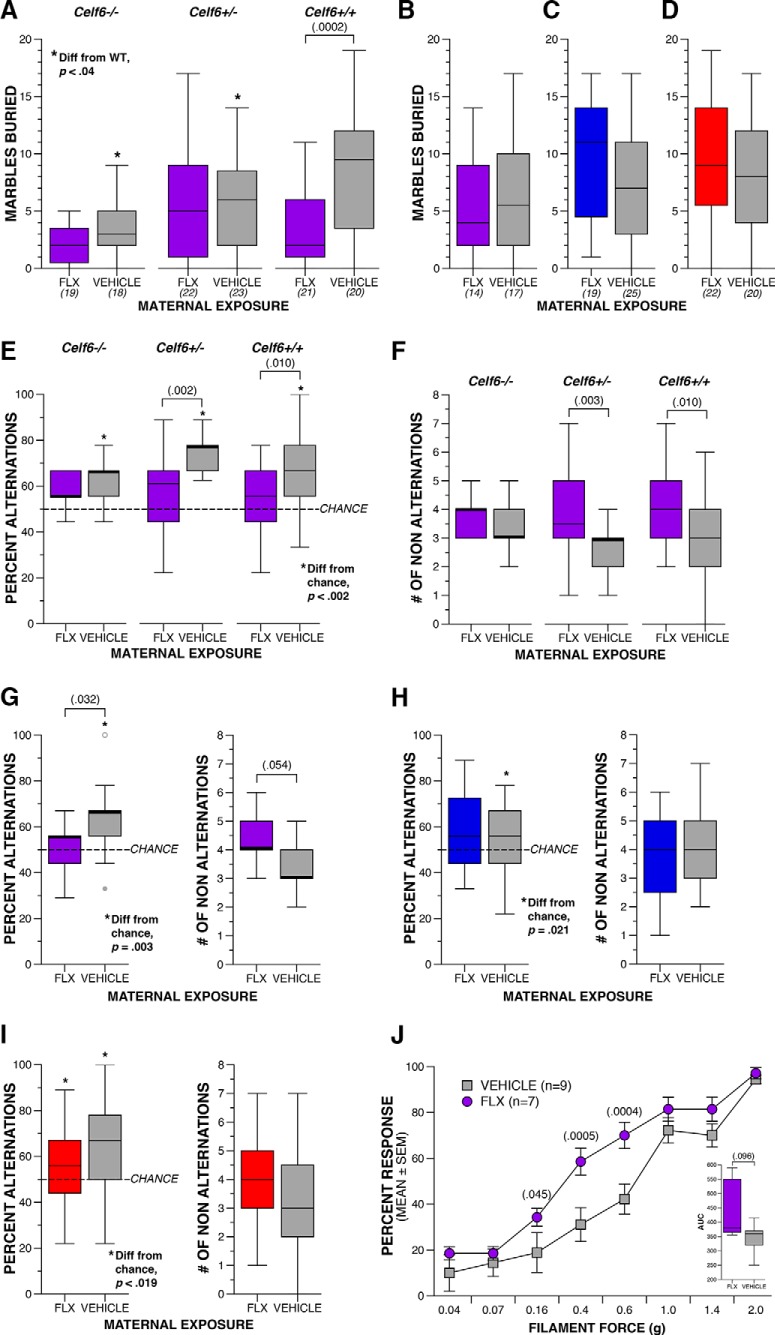
Extended maternal FLX induces repetitive, restricted patterns of behavior and tactile hypersensitivity. ***A***, Boxplot of number of marbles buried by *Celf6*-Extended FLX and VEH *Celf6* mutant and WT littermates during adulthood (genotype × drug interaction, *p* = 0.032; * denotes significant difference from WT littermates at *p* < 0.04 within VEH-exposed mice). ***B–D***, Boxplots of number of marbles buried by C57-Extended (***B***; drug, *p* = 0.303), Long Prenatal (***C***; drug, *p* = 0.234), and Short Prenatal (***D***; drug, *p* = 0.684) FLX and VEH C57BL/6J mice. ***E***, ***F***, Boxplots of percentage alternation trials (***E***; * denotes significant difference from chance at *p* < 0.002) and number of non-alternation trials (***F***) in the spontaneous alternation T-maze for *Celf6*-Extended FLX and VEH *Celf6* mutant and WT littermates (drug, *p* = 0.0001). ***G–I***, Boxplots of percentage alternation trials and number of non-alternation trials C57-Extended (***G***; * denotes significant difference from chance at *p* = 0.003), Long Prenatal (***H***; * denotes significant difference from chance at *p* = 0.021), and Short Prenatal (***I***; * denotes significant difference from chance at *p* < 0.019) C57BL/6J FLX and VEH mice. ***J***, Percentage of trials during which a response was elicited by von Frey filament presentation for C57-Extended FLX and VEH C57BL/6J mice (data are mean ± SEM; filament × drug, *p* = 0.005). Inset boxplot represents total AUC for all filaments per drug group. For boxplots, thick horizontal lines signify respective group medians, boxes are 25th–75th percentiles, whiskers are 1.5 × IQR, closed and open circles depict outliers.

Mice compulsively dig in bedding, and this behavior is perturbed in models of obsessive-compulsive disorder and ASD (Angoa-Pérez et al., 2013). Therefore, we examined digging in our mice using buried marbles as a proxy for compulsive digging. In the *Celf6*-Extended cohort, *Celf6* genotype alone decreased compulsive digging (*p* = 0.001^pp^). In addition, FLX treatment reduced digging in *Celf6* ^+/+^ mice (drug × genotype interaction, *p* = 0.032^qq^; *Celf6* ^+/+^ mice only, *p* = 0.0002^rr^; Fig. 5*A*). However, this effect on WT mice did not replicate in the C57-Extended cohort (*p* = 0.303^ss^; Fig. 5*B*). The reason behind this lack of replication is unclear. While the *Celf6* mice were backcrossed for many generations on to the C57BL/6J strain, it is possible there are subtle effects due to genetic drift in the *Celf6* colony. In the Long Prenatal and Short Prenatal cohorts, no difference in number of buried marbles was observed (*p* = 0.234^tt^ and *p* = 0.684^tt^, respectively; Fig. 5*C*,*D*). These data suggest that postnatal, but not prenatal, FLX may influence compulsive digging, but the impact of background strain on this effect requires further examination.

In spontaneous alternation in the T-maze, we examined whether the mice alternated which arm was explored between consecutive trials at a rate greater than chance (50%), which would suggest the mice demonstrated a typical exploration pattern and did not perseverate. We also examined if this percentage alternation was different between groups to understand if there was an effect of maternal FLX exposure on typical exploration patterns. We observed differences in the effect of FLX depending on whether exposure was prenatal only or extended postnatally. Extended FLX exposure induced perseverative behavior in this task as observed through percentage alternations that were no different from chance in the FLX-exposed mice. VEH mice from both *Celf6*-Extended and C57-Extended cohorts showed percentage of alternations better than chance (*p* < 0.002^uu^ and *p* = 0.003^vv^, respectively; Fig. 5*E*,*G*), and greater than that exhibited by FLX-exposed mice (*p* = 0.0001^ww^ and *p* = 0.032^xx^; Fig. 5*E*,*G*). This is also reflected in an increased number of non-alternations in FLX mice (*Celf6*-Extended main effect of drug, *p* = 0.0001^yy^; *Celf6* ^+/-^, *p* = 0.003; *Celf6* ^+/+^, *p* = 0.010; Fig. 5*F*, and a trend in the C57-Extended cohort, *p* = 0.054^zz^; Fig. 5*G*). In contrast, Long and Short Prenatal exposure to FLX did not result in percentage alternations different from VEH mice or increased non-alternation trials (*p* = 0.706^aaa^, *p* = 0.220^bbb^, *p* = 0.214^ccc^, and *p* = 0.220^ddd^, respectively; Fig. 5*H*,*I*). While in the Long Prenatal cohort VEH mice exhibited a percentage alternation trials greater than chance (*p* = 0.021^eee^) and FLX-exposed mice did not (*p* = 0.108^fff^), both VEH and FLX mice of the Short Prenatal cohort alternated at a percentage greater than chance (*p* < 0.020^ggg^) These results suggest that extended FLX exposure is required to induce perseverative behavior.

### Maternal FLX results in tactile hypersensitivity

Because we observed abnormalities in marble burying and T-maze performance only in the Extended exposure cohorts, we further examined FLX influence in this cohort on the sensory reactivity aspect of the restricted and repetitive behavior symptom domain. Previously, tactile processing defects were observed in the *Mecp2* and *Gabrb3* models of ASD (Orefice et al., 2016). We, therefore, tested tactile sensitivity using the von Frey filaments in a subset of the C57-Extended mice and observed hypersensitivity to tactile stimulation: FLX resulted in an increased percentage of trials with a response to stimulation compared to VEH mice (*p* = 0.005^hhh^; Fig. 5*J*) for filaments providing 0.16–0.6 g of force (*p* < 0.046^iii^). AUC was also greater for FLX compared to VEH mice (*p* = 0.096^jjj^), although it did not reach statistical significance, indicating a trend for a greater overall response to stimulation across filaments that likely requires a better-powered sample to observe significance. This tactile hypersensitivity is independent of general activity levels, altered emotionality (anxiogenic behavior), or sensorimotor abilities, as we did not observe differences between Extended FLX and VEH exposure in a 1-h locomotor activity task (distance traveled, center zone time and entries) or on a battery of sensorimotor tasks assessing balance, strength, and coordination (data not shown).

### Adult FLX treatment partially rescues tactile hypersensitivity yet exacerbates dominance phenotype induced by maternal FLX exposure

**Figure 6.**
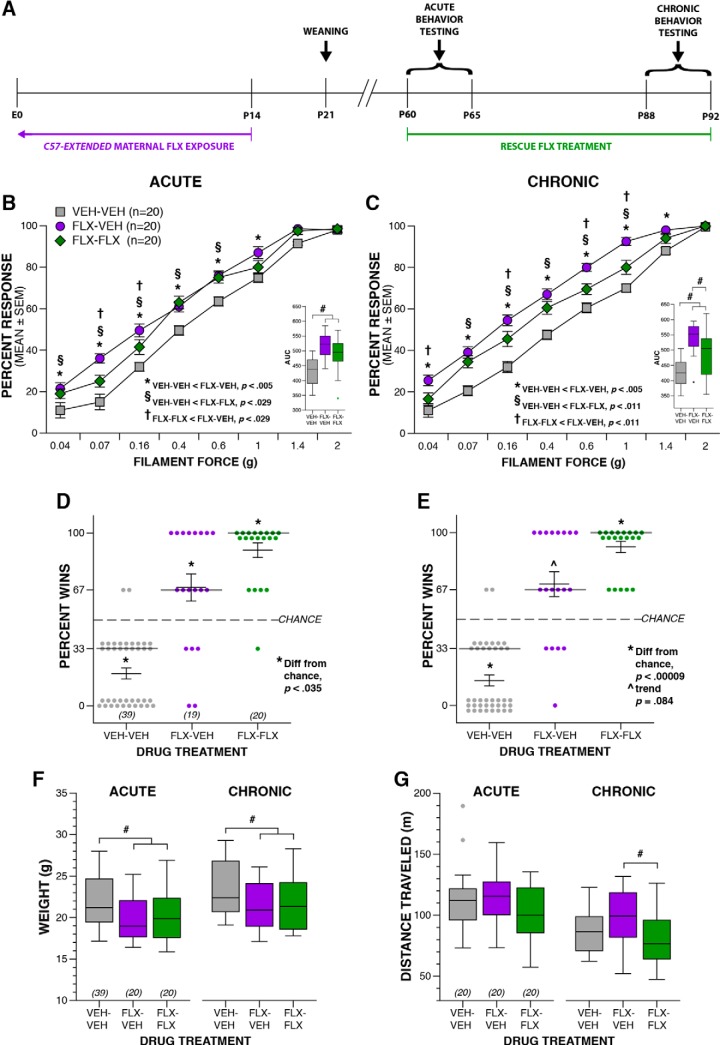
Re-exposure with FLX in adulthood ameliorates tactile hypersensitivity but increases dominance following maternal FLX exposure. ***A***, Schematic of treatment paradigm for maternal FLX exposure and behavioral testing following acute and chronic re-exposure with FLX in adulthood. ***B***, ***C***, Percentage of trials during which a response was elicited by von Frey filament presentation for Rescue VEH-VEH, VEH-FLX, and FLX-FLX C57BL/6J mice following acute (***B***; filament × drug, *p* = 0.002) and chronic (***C***; filament × drug, *p* < 0.000005) FLX re-exposure (data are mean ± SEM; * denotes significant difference between VEH-VEH and FLX-VEH; § denotes significant difference between VEH-VEH and FLX-FLX; † denotes significant difference between FLX-FLX and FLX-VEH). Inset boxplot represents total AUC for all filaments per drug group. ***D***, ***E***, Dot plots of percentage of wins during tube test of social dominance between VEH-VEH and VEH-FLX, and between VEH-VEH and FLX-FLX adult mice in the Rescue cohort following acute (***D***; * denotes significant difference from chance at *p* = 0.035) and chronic re-exposure (***E***; * denotes significant difference from chance at *p* < 0.00009; ^ denotes marginally significant difference from chance at *p* = 0.084). Crosshairs represent mean ± SEM, and dark gray lines represent medians. ***F***, Boxplots of weight of Rescue VEH-VEH, VEH-FLX, and FLX-FLX C57BL/6J mice following acute (drug, *p* < 0.000005) and chronic (drug, *p* < 0.000005) FLX re-exposure. ***G***, Boxplots of distance traveled during the 1-h locomotor activity test by Rescue VEH-VEH, VEH-FLX, and FLX-FLX C57BL/6J mice following acute (drug, *p* = 0.070) and chronic (drug, *p* < 0.020) FLX re-exposure. For boxplots, thick horizontal lines signify respective group medians, boxes are 25th–75th percentiles, whiskers are 1.5 × IQR, closed and open circles depict outliers; # denotes significantly different Tukey’s *post hoc* comparison.

During brain development, 5-HT regulates the development of its own system through a negative feedback mechanism (Whitaker-Azmitia et al., 1996). Studies have shown persistent alterations to the 5-HT system in adults following developmental SSRI exposure through the dam, including increased 5-HT1a receptor sensitivity, and decreased 5-HT transporter expression, Tph2 levels in the dorsal raphe, and midbrain 5-HT content (Cabrera-Vera et al., 1997; Maciag et al., 2006; Noorlander et al., 2008; Olivier et al., 2011). These findings suggests a disrupted 5-HT system may be mediating the long-term behavioral disruptions in our mice. Indeed, adult alterations to 5-HT activity have been shown to produce similar phenotypes. Tryptophan-depleted diets increased social dominance in the adult mouse (Uchida et al., 2005) and spontaneous alternation rates in adult rats (González-Burgos et al., 1995). Mice null for Lmx1b, a transcription factor required for differentiation of postmitotic 5-HT neurons, lack central 5-HT and showed reduced responsiveness to von Frey filaments (Zhao et al., 2007). These studies indicate a link between disrupted 5-HT levels and social dominance, alternation rates, and tactile sensitivity. Acute FLX treatment has been shown to increase extracellular 5-HT levels (Malagié et al., 1995), while chronic treatment (lasting at least three weeks) may actually reduce 5-HT levels through autoreceptor feedback and reduced transporter-mediated 5-HT recycling (Siesser et al., 2013; Bazhenova et al., 2017). However, the literature on this is inconsistent (Jacobsen et al., 2016). To determine whether altering levels of 5-HT through SSRI treatment can rescue the behavioral deficits we observed in maternally-exposed pups, we treated an independent cohort of C57-Extended mice (Rescue cohort) with FLX through drinking water starting at P60 and examined their behavior following acute (<5 d) and chronic (more than three weeks) treatment (Fig. 6*A*). Output from statistical tests for this section is fully reported in Table 6.

**Table 6.** Statistical summary for Figure 6

Variable		Comparison	Data structure	Statistical test	Output	*p* value	*Post hoc* power	Effect size
Percent response trials	kkk	ACUTE Rescue, drug × filament interaction	Non-normal	One-way rmANOVA	*F*_(6.158,351.02)_ = 2.619	*p* = 0.002	0.981	0.303
	lll	0.04 g filament, drug	Non-normal	Simple main effect	*F*_(2,456)_ = 4.543	*p* = 0.011	0.772	0.139
	lll	0.07 g filament, drug	Non-normal	Simple main effect	*F*_(2,456)_ = 16.661	*p* < 0.000005	1	0.270
	lll	0.16 g filament, drug	Non-normal	Simple main effect	*F*_(2,456)_ = 11.590	*p* = 0.00001	0.994	0.225
	lll	0.4 g filament, drug	Non-normal	Simple main effect	*F*_(2,456)_ = 8.016	*p* = 0.0004	0.956	0.185
	lll	0.6 g filament, drug	Non-normal	Simple main effect	*F*_(2,456)_ = 7.286	*p* = 0.0008	0.936	0.179
	lll	1.0 g filament, drug	Non-normal	Simple main effect	*F*_(2,456)_ = 5.487	*p* = 0.004	0.849	0.157
	mmm	ACUTE Rescue AUC, drug	Normal	One-way ANOVA	*F*_(2,57)_ = 15.887	*p* < 0.000005	0.999	0.747
	nnn	CHRONIC Rescue, drug × filament interaction	Non-normal	One-way rmANOVA	*F*_(6.665,13.33)_ = 4.506	*p* < 0.000005	1	0.398
	ooo	0.04 g filament, drug	Non-normal	Simple main effect	*F*_(2,456)_ = 8.840	*p* = 0.0001	0.971	0.196
	ooo	0.07 g filament, drug	Non-normal	Simple main effect	*F*_(2,456)_ = 15.357	*p* < 0.000005	0.999	0.259
	ooo	0.16 g filament, drug	Non-normal	Simple main effect	*F*_(2,456)_ = 21.158	*p* < 0.000005	1	0.305
	ooo	0.4 g filament, drug	Non-normal	Simple main effect	*F*_(2,456)_ = 16.264	*p* < 0.000005	1	0.266
	ooo	0.6 g filament, drug	Non-normal	Simple main effect	*F*_(2,456)_ = 15.714	*p* < 0.000005	0.999	0.261
	ooo	1.0 g filament, drug	Non-normal	Simple main effect	*F*_(2,456)_ = 20.966	*p* < 0.000005	1	0.303
	ooo	1.4 g filament, drug	Non-normal	Simple main effect	*F*_(2,456)_ = 4.179	*p* = 0.016	0.735	0.135
	ppp	CHRONIC Rescue AUC, drug	Normal	One-way ANOVA	*F*_(2,57)_ = 20.307	*p* < 0.000005	1	0.844
Percent alternating trials	qqq	ACUTE Rescue, drug	Normal	Two-way ANOVA	*F*_(2,54)_ = 1.766	*p* = 0.181	0.354	0.255
	rrr	CHRONIC Rescue, drug	Non-normal	Two-way ANOVA	*F*_(2,51)_ = 0.814	*p* = 0.449	0.182	0.179
Percent tube test bouts won	sss	ACUTE Rescue VEH-VEH, compared to 50%	Non-normal	One-spl Wilcoxon	*Z* = -5.312	*p* < 0.000005	N/A	0.74
	ttt	ACUTE Rescue FLX-VEH, compared to 50%	Non-normal	One-spl Wilcoxon	*Z* = 2.114	*p* = 0.034	N/A	0.25
	vvv	ACUTE Rescue FLX-FLX, compared to 50%	Non-normal	One-spl Wilcoxon	*Z* = 4.016	*p* = 0.00006	N/A	0.85
	sss	CHRONIC Rescue VEH-VEH, compared to 50%	Non-normal	One-spl Wilcoxon	*Z* = -5.533	*p* < 0.000005	N/A	0.81
	uuu	CHRONIC Rescue FLX-VEH, compared to 50%	Non-normal	One-spl Wilcoxon	*Z* = 1.726	*p* = 0.084	N/A	0.17
	vvv	CHRONIC Rescue FLX-FLX, compared to 50%	Non-normal	One-spl Wilcoxon	*Z* = 3.934	*p* = 0.00008	N/A	0.81
Weight	www	ACUTE Rescue, drug	Normal	Two-way ANOVA	*F*_(2,73)_ = 15.468	*p* < 0.000005	0.999	0.652
	www	CHRONIC Rescue, drug	Non-normal	Two-way ANOVA	*F*_(2,73)_ = 18.850	*p* < 0.000005	1	1.814
Distance traveled	xxx	ACUTE Rescue, drug	Normal	Two-way ANOVA	*F*_(2,54)_ = 2.787	*p* = 0.070	0.526	0.322
	yyy	CHRONIC Rescue, drug	Normal	Two-way ANOVA	*F*_(2,54)_ = 7.742	*p* = 0.020	0.713	0.378

Effect size for *F* tests reported as Cohen’s *f* (Cohen, 1988; interpretation: 0.01 = small; 0.25 = medium; 0.40 = large) and for nonparametric tests reported as η^2^.

Re-exposure with FLX influences tactile sensitivity and social dominance phenotypes induced by maternal FLX exposure, but likely through different mechanisms. The tactile hypersensitivity observed in adult mice exposed to maternal FLX was partially rescued by re-exposure with FLX. No drug × sex interaction was observed, therefore data are reported collapsed by sex. We replicated in both acute and chronic testing the tactile hypersensitivity observed in the C57-Extended cohort. During acute re-exposure, differences in tactile responsiveness were observed between treatment groups (*p* = 0.002^kkk^) for all but the largest two filaments that elicit near 100% response from all mice (*p* < 0.012^lll^; Fig. 6*B*), For each of these filaments, the FLX-VEH group exhibited increased percentage of trials with a response to stimulation compared to VEH-VEH mice while the FLX-FLX mice began to show a reduction in responsiveness to presentation of the von Frey filaments providing 0.07–0.16 g of force compared to FLX-VEH mice. The overall response to stimulation as measured by AUC was not different between FLX-treated groups, although each was greater than that for the VEH-VEH mice (*p* < 0.000005^mmm^). After three more weeks of FLX treatment, differences between treatment groups (*p* < 0.000005^nnn^) were now observed for all filaments except the largest, for which all mice responded 100% of the time (*p* < 0.017^ooo^; Fig. 6*C*). The FLX-FLX mice showed further reduction in responsiveness compared to the FLX-VEH group for filaments providing 0.04, 016, 0.6, and 1.0 g of force. Analysis of the AUC revealed the overall responsiveness for the FLX-FLX group was now significantly lower than the FLX-VEH group (*p* < 0.000005^ppp^). These data suggest the tactile hypersensitivity induced by maternal FLX exposure can be alleviated by FLX treatment, further supporting a role for the 5-HT system in the circuitry mediating this phenotype.

Despite observing increased perseverative behavior in the spontaneous alternation T-maze for FLX-exposed mice in both the *Celf6*-Extended and C57-Extended cohorts, we did not replicate this baseline difference in phenotype a third time in the Rescue cohort. During acute and chronic testing, no differences were observed between drug groups for percentage of alternations (*p* = 0.181^qqq^ and *p* = 0.449^rrr^, respectively; Table 5) or number of non-alternation trials (data not shown). Thus, it remains unclear if this phenotype, when present, would be reverted by adult FLX treatment.

Surprisingly, the enhanced dominance phenotype observed in mice exposed to maternal FLX was exacerbated by both acute and chronic FLX re-exposure. In the Rescue cohort, the increased dominance observed in the C57-Extended cohort was replicated. The VEH-VEH mice lost more bouts compared to chance (50%) during both acute and chronic testing (*p* < 0.000005^sss^; Fig. 6*D*,*E*), while FLX-VEH mice won more bouts compared to chance, although this failed to reach statistical significance during chronic treatment testing (*p* = 0.034^ttt^ and *p* = 0.084^uuu^). The FLX-FLX group also displayed increased dominance by winning more bouts than expected by chance during both acute and chronic testing (*p* < 0.00009^vvv^; Fig. 6*D*,*E*). The mean and median differences suggest that the FLX re-exposure further increased the dominance behavior in mice exposed to maternal FLX (acute: FLX-VEH, *M* = 68.47, *Mdn* = 67, *SD* = 34.25; FLX-FLX, *M* = 90.05, *Mdn* = 100, *SD* = 19.01; chronic: FLX-VEH, *M* = 69.89, *Mdn* = 66, *SD* = 31.33; FLX-FLX, *M* = 91.50, *Mdn* = 100, *SD* = 15.10). As in the previous cohorts, the dominance phenotypes were not due to increased animal size in the FLX groups, as each actually weighed less than the VEH-VEH group (*p* < 0.000005^www^; Fig. 6*F*), with no change between acute and chronic treatment. We also examined the Rescue cohort in the 1-h locomotor activity task to determine whether the behavioral changes observed were due to general differences in activity levels or anxiogenic behavior induced by FLX re-exposure. We found a trend toward a decrease in total distance traveled in the FLX-FLX mice compared to the FLX-VEH mice during acute exposure (*p* = 0.070^xxx^; Fig. 6*G*) that reached statistical significance following chronic exposure (*p* = 0.020^yyy^), with no differences in center area variables suggesting no change in anxiety-related behavior (data not shown). ANCOVA with litter size as the covariate yielded a marginally significant effect of drug on distance traveled during chronic re-exposure testing (*p* = 0.072). We do not interpret these data as hypoactivity in the FLX-FLX group as their activity levels were not different from VEH-VEH mice nor were the FLX-VEH mice hyperactive compared to the control group in any cohort examined. It is possible there is a very small effect of FLX re-exposure on activity that we were underpowered to detect, but which likely does not confound the interpretation of the von Frey assessment or dominance phenotypes. In sum, the results from the Rescue cohort suggest disrupting 5-HT levels during development influenced the role of the 5-HT system in the behavioral circuits responsible for responses to sensory and social stimuli in the von Frey assessment of tactile sensitivity and the tube test of social dominance, respectively. Remarkably, the effects are in the opposite directions, suggesting they are mediated by distinct mechanisms. Specifically, SSRI treatment ameliorated the hypersensitivity to sensory stimuli but further exacerbated the response to social stimuli.

## Discussion

The widespread roles of 5-HT in neurodevelopmental processes are well-described (Sodhi and Sanders-Bush, 2004; Whitaker-Azmitia, 2010), and 5-HT dysregulation in a subset of patients with ASD has been well-documented and often replicated (McDougle et al., 1996, 1993; Chugani et al., 1999, 1997; Hollander et al., 2005; Azmitia et al., 2011; Benza and Chugani, 2015). Here, we examined the behavioral impact of *in utero* exposure to drugs that impact the 5-HT system. Human epidemiological studies suggest antidepressant use during pregnancy may increase ASD risk in offspring, although challenges remain in adjusting for maternal diagnosis appropriately. With current epidemiological samples, only some analyses confidently demonstrated an effect of SSRI treatment independent of maternal diagnosis, although most were not inconsistent with modest additional risk attributable to treatment. Given these challenges interpreting the epidemiological studies in aggregate, we tested the hypothesis that maternal SSRI exposure, independent of maternal stress, can modulate ASD-relevant behaviors in mammals. We report social communication and interaction deficits, as well as repetitive patterns of behavior in offspring of dams exposed to the SSRI FLX during pre- and postnatal development. We further showed that re-exposure with FLX can ameliorate tactile hypersensitivity, yet further shift social dominance behaviors. These findings indicate that in the absence of other maternal manipulations or stressors, drug exposure alone is sufficient to induce in offspring long-term consequences to social and restrictive behaviors, some of which may be mediated by a disrupted 5-HT system.

There is an established body of work in the rodent literature showing clear links between maternal SSRI exposure during pregnancy and a paradoxical increase in depressive- and anxiety-like behaviors in the mature offspring (Lisboa et al., 2007; Noorlander et al., 2008; Olivier et al., 2011; Avitsur et al., 2016; Boulle et al., 2016; Gobinath et al., 2016; Salari et al., 2016), but little analysis of the impact on social or repetitive behavioral circuits. The current study adds to the limited studies of dam SSRI exposure that have recently begun to focus on these types of behaviors in offspring, and is the first to fully characterize in this type of model behaviors relevant to the core symptoms of ASD, including multiple tasks within each distinct domain. We sought to examine in our mice various possible social disruptions and repetitive/restricted behaviors, including sensory sensitivities, that are observed in autistic individuals. We demonstrate the potential for maternal SSRI exposure alone to induce early social communication deficits, abnormal sociability, and altered social hierarchy behaviors, as well as perseveration and tactile hypersensitivity.

We did not find any phenotype common among all three exposure durations, suggesting FLX’s influence on ASD-related behaviors may depend on the duration of and developmental timeframe of exposure. Early pregnancy alone (E0–E16) was the least vulnerable developmental period examined. We observed increased submissive behaviors in adults in this exposure model, but typical behaviors in all other testing. Increased submissive behaviors were also observed in adult offspring that received FLX exposure through the entirety of gestation, or the rough equivalent in brain development to the first two trimesters of human pregnancy. In addition, this increased exposure duration induced early communicative deficits in the form of reduced USV production when isolated from the dam, as well as sociability disruptions. The Extended FLX exposure groups exhibited the greatest functional disruptions. The dampened USV production during development was coupled with social approach decreases and robust dominance behaviors suggesting this longer duration exposure to altered 5-HT activity most heavily impact social behavior circuitry. Only these mice demonstrated repetitive/restricted patterns of behavior. Complementing our findings on distinct effects of maternal FLX on dominance, recent work showed prenatal maternal FLX treatment decreased aggressive behaviors, while treatment extending postnatally increased aggressive behaviors in adult C57BL/6 male offspring (Kiryanova et al., 2016). However, another report showed increased aggression in male offspring of ICR dams exposed to only prenatal FLX (Svirsky et al., 2016). The discrepancies in aggression findings between these two studies may reflect strain × drug interactions. The distinct phenotypes of mice that received prenatal-only versus continued postnatal FLX exposure may be mediated by circuitry disruptions due to differences in 5-HT system development that occurs at these different periods. While 5-HT axons reach their targets by birth, terminal field development occurs postnatal (Maddaloni et al., 2017). Excess 5-HT during embryonic development acts to down-regulate 5-HT innervation through a negative feedback mechanism (Whitaker-Azmitia, 2005) and reduced 5-HT terminal processes has also been reported in rodents following postnatal SSRI treatment (Maciag et al., 2006). This suggests prenatal FLX exposure likely influences axonal innervation by 5-HT neurons of the raphe, but continued postnatal exposure may have further reduced 5-HT terminal fields, possibly meditating the increased dominance and perseverative behavior patterns observed in the Extended FLX cohort.

The social behavior disruptions observed in our study extend those previously reported following maternal FLX exposure to include sociability alterations and strong influences on social dominance. The majority of previous examinations of early SSRI exposure on social phenotypes focused only on aggression (Lisboa et al., 2007; Kiryanova et al., 2016; Svirsky et al., 2016), as a clear link between 5-HT and aggression has been shown in both humans and animal models (Brown et al., 1982; Kaplan et al., 1994; Moeller et al., 1996; Reisner et al., 1996). We found sociability was dampened following FLX during pregnancy only, and that extended FLX exposure decreased total time investigating the social stimulus, but did not disrupt a social preference. This suggests we are observing a differential impact on sociability circuits based on timing of exposure. A previous report showed maternal FLX exposure limited to postnatal-only ages (P3–P21) had no effect on social approach behaviors in mice (Nakai et al., 2017). Together with our findings, this suggests *in utero* exposure may be the vulnerable period for sociability circuit formation. Tryptophan depletion diet has been shown to disrupt sociability behavior in adult C57BL/6 mice (Zhang et al., 2015). It is possible that early FLX exposure ultimately decreases 5-HT activity in key areas that mediate social preference. We did not find an influence of extended exposure on frequency of social behaviors observed during the juvenile interactions. Whether this is a result of the age at testing or that the unexposed partner could also initiate the interactions is unknown, and we are unaware of another study investigating unimpeded social interactions in a similar model.

Perhaps the most robust phenotype we observed was the change to social dominance. This is unsurprising given a link between low 5-HT levels in the mature brain and dominance has been demonstrated in both human and animal research (Kaplan et al., 1994; Uchida et al., 2005). Tryptophan depletion was shown in an adult autistic patient to exacerbate symptoms including perseveration (McDougle et al., 1993), and adult mice fed a tryptophan-depleted diet exhibited increased dominance in the tube test (Uchida et al., 2005). If a decrease in activity of the 5-HT system is mediating the dominance phenotypes observed in our mice, then we hypothesized increasing this activity would normalize this phenotype. Interestingly, we observed the opposite. Re-exposure with FLX during adulthood actually further enhanced the dominant phenotype induced by maternal FLX exposure. Within 30 min of exposure FLX increases extracellular 5-HT, dose-dependently, within the frontal cortex, hippocampus, and raphe (Malagié et al., 1995). Cortical and striatal 5-HT tissue levels are depleted with chronic (three weeks) exposure (Siesser et al., 2013; Bazhenova et al., 2017), but extracellular 5-HT levels seem to remain elevated (Jacobsen et al., 2016). Our data suggest the maternal FLX exposure altered the circuits mediating this social hierarchy behavior in a complex manner such that they no longer respond to 5-HT in a typical way. It is possible that other aspects of the 5-HT system, such as receptor densities or innervation patterns, were altered by maternal FLX exposure such that adult FLX treatment influences these circuits differently.

In addition to social behavior disruptions, maternal FLX exposure induced abnormalities related to the repetitive/restricted patterns of behavior symptoms of ASD. Our results suggest continued postnatal exposure may be required to perturb these circuits. Most robust was the induction of tactile hypersensitivity. Sensory processing dysfunction is associated with multiple neurodevelopmental disorders, including the sensory sensitivity observed in ASD (Slobounov et al., 2006; Ross et al., 2007; Schneider et al., 2008; Lane et al., 2010; Ghanizadeh, 2011; Dar et al., 2012). 5-HT appears to play a role in regulating the balance between internal signals and sensory information from the environment (Lottem et al., 2016). Thus, fluctuations in the 5-HT system could tip that balance creating increased or decreased sensitivities. The high levels of 5-HT required during neurodevelopment likely serve to increase the brain’s responsiveness to the environment at that time required for plasticity and maturation. Adjusting those levels as we did through maternal FLX treatment may disrupt the 5-HT-mediated sensitivity required for proper circuit development. Our results suggest the circuits underlying tactile sensitivity were altered, perhaps made hyper-responsive. The partial rescue of the tactile hypersensitivity observed following adult FLX re-exposure suggests the circuit disruptions are reversible and may be due to abnormal 5-HT activity levels. These data further support the therapeutic potential of SSRIs for sensory processing disorders.

As genetic factors are clearly an important causation of ASD (Geschwind, 2008), it is likely that environmental contributions to ASD risk interact with existing genetic susceptibility (Hertz-Picciotto et al., 2006; Klei et al., 2012). It has been suggested that environmental factors that might modulate social behavior or language could tip the balance toward ASD in children with genetic vulnerability (Geschwind, 2008). As we initially thought SSRI exposure alone might be a relatively modest factor, we exposed *Celf6* mutant mice, which exhibit a subtle ASD-like phenotype (Dougherty et al., 2013), to maternal FLX and analyzed offspring behavior for possible potentiation of the ASD-like phenotype. We hypothesized a potentiation of deficits in *Celf6* mutants with FLX exposure due to the effect each manipulation has on the 5-HT system. The *Celf6* mutant exhibited subtle ASD-related deficits, specifically decreased early social communicative behavior and a resistance to change behavior patterns as well as reduced brain 5-HT levels patterns (Dougherty et al., 2013), making it an ideal model to examine the influence of FLX on a genetically vulnerable background and the impact of two hits to the 5-HT system on these behaviors. What we observed was both the *Celf6* mutation and the FLX exposure independently reduced pup USVs, induced perseveration in the T-maze and reduced digging in the marble burying assay. These complementary behavioral disruptions suggest Celf6 loss and FLX exposure act in parallel on the circuits underlying these behaviors, possibly through similar influences on the 5-HT system. In contrast to our results is a similar study in the 15q11-13 duplication model (15q-dup), which also shows reduced brain 5-HT levels (Tamada et al., 2010). Interactions between maternal FLX and the genetic duplication potentiated deficits in the 15q-dup mice: specifically, hypoactivity and anxiogenic behaviors (Nakai et al., 2017). Maternal FLX actually improved 15q-dup induced sociability, which was linked to restoration of extracellular 5-HT levels. The effect of FLX on the development of behavior circuitry appears to be in the opposite direction to that induced by 15q-dup, such that it can have an ameliorating effect. However, the effect is likely in the same direction as *Celf6* loss and similar enough that the effects are paralleled and not synergistic. Thus, we did not observe a potentiation of behaviors in the FLX-exposed *Celf6* mutants or a restoration of deficits.

It is unclear why the Extended-exposure *Celf6* ^+/+^ mice, which are on a C57BL/6J background, behaved differently than the C57BL/6J mice in marble burying. No difference was observed between the *Celf6* ^+/+^ and C57 VEH-exposed mice, indicating the source of this difference is the maternal FLX exposure groups. A similar phenomenon was described in the Neuroligin-3 knock-out mice showing deficits in sociability in both knock-out and WT littermates when housed together (Kalbassi et al., 2017). It is possible the reduced digging behavior expressed by the *Celf6* ^-/-^ mice influences that of their WT littermates following maternal exposure to FLX. An alternative explanation is that perhaps digging behavior was influenced by the maternal behavior of *Celf6^+/-^* versus C57BL/6J dams. The focus of this study was on the long-term consequences of exposure on offspring behavior, but we cannot rule out that some of our results may be influenced by SSRI mediated alterations of maternal behaviors in the nest. We chose not to cross-foster our pups because we wanted to continue FLX exposure into postnatal stages of brain development, and we wanted to avoid the confounding stress to both pups and dams of direct pup injections. Yet, because of this design, we cannot comment on the influence of FLX on maternal behavior in our litters, nor any long-term effects of maternal behavior changes on adult offspring phenotypes.

The potential influence of maternal care is complex, and worthy of an entire study of its own. Qualitatively, differences in maternal care have not been observed in *Celf6^+/-^* dams, yet this has not been thoroughly quantified. In addition, there may be an interaction between direct FLX exposure and heterozygous loss of *Celf6* that affects maternal behavior and maternal care. The reciprocal influence of maternal care and pup USV on each other is complex. Greater maternal responsiveness has been shown to result in fewer calls emitted by the pups (D’Amato et al., 2005). However, decreased USV production by pups has also been shown to result in maternal neglect because the dams cannot locate the pups outside of the nest. This was identified in vocally impaired pups with genetic loss of motor neurons that transform breaths into calls (Hernandez-Miranda et al., 2017). It is possible in our FLX model that pup USV and maternal care are interacting in several ways. First, FLX could be directly impacting maternal care, and decreasing pup USVs. If this is the case in our mice, we would hypothesize based on previous research that the FLX increased maternal care and thus reduced pup USVs. We would further hypothesize this level of maternal care would likely not result in the long-term behavioral deficits observed in the adult offspring. However, the large magnitude of the reduction in USV we observed in FLX-exposed pups seems too robust for changes in maternal care to account for the underlying the pup phenotype. A second way in which maternal care and pup USV may be interacting is through a reduction to maternal care in response to the robustly reduced USV emitted by the pups exposed to FLX. This reduced maternal care has the potential to further disrupt neurodevelopment of the pup, and thus be a possible indirect influence on the later adult behaviors. *Celf6* mutation harbored by the dam may also play into this scenario by altering dam or pup responses additively or synergistically. To our knowledge, the direct impact of SSRI exposure on maternal behaviors has not been examined; however increased latency to retrieve pups back to the nest has been demonstrated in adult female offspring exposed gestationally to FLX (Svirsky et al., 2016), suggesting transgenerational effects of gestational FLX exposure. Thus, we can conclude that FLX treatment to the dam during and immediately following pregnancy modulates progeny behaviors relevant to ASD; and that this is independent of maternal stress but possibly mediated by alterations to maternal care behaviors.

Despite a potential for increased risk from FLX exposure, untreated or undertreated depression and anxiety in pregnancy are themselves strongly associated with adverse outcomes (Grote et al., 2010), and we do not view this study as being sufficient cause to alter treatment decisions. Thus, while our findings are a contribution to our understanding of the consequences of developmental SSRI exposure, additional work needs to be done to understand the precise mechanisms by which SSRIs can alter circuit function. Our rescue experiment indicates that tactile sensitivity may be responsive to restoring 5-HT levels via SSRI treatment, but that this could exacerbate other phenotypes. This also indicates these two phenotypes have distinct mechanisms. We believe the carefully characterized phenotype demonstrated here provides a clear paradigm for comparative analysis of different treatment options for their relative impact on offspring behavior, as well as a potential experimental manipulation for studies defining the circuits that control social and repetitive behaviors in the mammalian brain.

## Synthesis

Reviewing Editor: Orly Reiner, Weizmann Institute of Science Decisions are customarily a result of the Reviewing Editor and the peer reviewers coming together and discussing their recommendations until a consensus is reached. When revisions are invited, a fact-based synthesis statement explaining their decision and outlining what is needed to prepare a revision will be listed below. The following reviewer(s) agreed to reveal their identity: Georgianna Gould.

The reviewers have indicated that the study is of interest and have provided constructive suggestions on how to improve the study.

This is a well-designed, well-powered and extensive study of the behavioral impact of antidepressant exposure during neurodevelopment. Importantly, three periods of exposure were analyzed, the longest exposure (referred to as ‘extended’) covering prenatal and early postnatal development in mice, thus approximating the entire duration of human pregnancy, including periods of nursing. Impact of antidepressant exposure was assessed in mice with a genetically vulnerable background for autism spectrum disorders (i.e. Celf6-/- mice), and heterozygous and wild-type littermates, allowing the authors: 1) to assess whether antidepressant exposure alone is sufficient to induce long-term behavioral alterations, and 2) to test whether antidepressant exposure in a vulnerable background could act synergistically and potentiate ASD phenotype. Rescue experiments on the ‘extended’ exposure cohort were also performed.

The study uses a mouse gestational fluoxetine exposure study with a higher than typical dose of drug (16 mg/kg/d) administered in drinking water sweetened with saccharin to dams (an unusual approach in an attempt to reduce stress) and also seeks to ameliorate deficits in offspring with fluoxetine treatment and a Celf6 mutant line of mice exhibiting mild autism-like behavioral phenotype along with wild type mice. Celf6 has been shown in other work to impact SERT function and to be associated with autism. The authors do not provide any neurochemical or analytical measures of serotonin levels or fluoxetine levels to confirm the effects of behavior are due to drug treatments.

The reviewers had very specific concerns, which are expected to be addressed during the revision process.

The statistical analyses appear for the most part to be correct. However there are a few concerns. First, if tests involve siblings from the same litter, one way to account for this factor is to include it as a covariate in any of the models. Also for figures it is not clear why social preferences are expressed in box plots with medians and inter-quartile ranges if they are parametric, then means SEM and SD would be far more conventional and also more clear for readers to understand.

Extended data:

Statistical tables are provided in text, they could alternately be placed in an appendix if more consistent with journal format.

Software comments:

1. Avisoft Recorder software for USV, with frequency sonograms prepared using MATLAB. No mention of validation of software.

2. MiceProfiler used for tracking both stimulus and experimental mice in tests of juvenile social interaction no validation but tracking was supervised and corrected manually, then MATLAB was used to process tracked videos. When frames were missing data points they were re-analyzed following smoothing.

3. Any-Maze software was used for social approach and 1-h locomotor activity, no validation tests or results were described.

Specific comments:

Overall a very interesting and potentially valuable manuscript examining in mice (wild-type and Celf6 mutant genotypes) the effects of 16 mg/kg/day fluoxetine exposure vs. vehicle during gestation with different stopping points for treatments on offspring behaviors relevant to autism. However since no measures of serum or brain fluoxetine or serotonin were made, it is not possible to know if the underlying mechanism is serotonin based. Further justification and explanation for the value added from use of Celf6 mouse genotypes or relevance to serotonergic mechanisms underlying the behaviors is lacking. Some revisions, not necessarily requiring further experiments, are required to render this manuscript acceptable for publication. Specific revisions or clarifications are as follows:

1. Introduction: The rationale for use of the Celf6 mutation mice and their clinical relevance to autism is not provided until the results section, and it really needs to be introduced earlier in the manuscript. On the other hand a the “meta-analysis” information appears as two run-on sentences and doesn't draw the reader in. Maybe this analysis belongs in the discussion instead?

2. In introduction, it is not clear what is meant by “acute deficits” and why they should be reversible, are the authors referring to 5-HT or SERT or behaviors?

3. Abstract, introduction and throughout manuscript: for acronyms such as “SSRI” or “CNS” or “OCD” on page 25 etc...authors should spell out in entirety at first mention and introduce acronym.

4. Check spelling and grammar throughout, and proofread revision carefully to find mistakes: for example authors probably mean “trophic” not “tropic” in abstract, and introduction second paragraph “over” appears without context in middle of sentence.

5.In the methods, was the Novalsan diluted at all, and if so by what %?

6. Social dominance: Are three bouts per mouse sufficient to establish its dominance?

7.What is the justification for the difference between “Long” and “Short” pre-natal? The distinction for this 4 day period seems arbitrary, so is there a basis for this distinction based on some critical biological change taking place during gestation?

8.The reduction in USV calls at postnatal days 5, 7, 9 measured appears to be the median number of calls per pup over 3 min. But it is not clear if same pups were measured repeatedly why these measures are not parametric. If parametric statistics were used then a repeated measures approach could be used. This measure appears to be highly variable, are outliers included or excluded from the analyses? Also given these differences, were fluoxetine exposed pups lost in the week following birth? There was some mention of smaller litter sizes in some fluoxetine treated groups.

9.Figure legends graphs and results text appear to be inconsistent with one another for adult social behavior and fig 3. For example, only figure D Celf6 -/- vehicle appear to have preference for interaction. While it states in the results that results are reported collapsed across sex, the fig 3 legend states a significant difference for female Celf6 mice after “D.” Also the authors report no significant differences, yet for E it appears that the vehicle treated mice might have a sociability preference, but this is not indicated by a star over bar.

10.Results p. 25 marble bury paragraph for fig 5, what do the authors mean by “cryptic genetic differences” may be present? Could this due to errors in genotyping, and how reliable is the genotyping method being employed? Could other factors be involved, aside from maternal care such as sex of subjects?

11. For the independent Rescue cohort, were these mice also tested as the other mice had been during juvenile development? Also dubbing this group “retreatment” seems a bit off the mark, since first exposure is gestational as only the mother is treated, and second exposure is the actual treatment to correct deficits in offspring.

12. The study lacks biochemical measures that could provide mechanistic insights. Were any measures of fluoxetine taken from maternal blood or brain or pup blood or brain to determine long term exposure levels?

13. In discussion the authors describe the study as having a “well-powered manner”, but specifically what they mean by this is not clear.

14. In references some of the titles are capitalized on all words while others only on first word, choose the format consistent with the journal and make sure it is used throughout.

Additional specific comments:

1) In the abstract, significance statement and introduction sections (and title), the authors do not clearly mention that the experiments were carried out in mice with a genetically vulnerable background for autism spectrum disorders (i.e. Celf6-/- mice).

Pg1, ln6: “Here, we sought to determine if maternal SSRI treatment alone, independent of maternal stress, as sufficient to induce in the offspring behavior disruptions relevant to ASD.”

Pg, ln5: “Our mouse studies show that, in the absence of other maternal manipulations or stressors, developmental SSRI exposure alone can alter the long-term functioning of behavioral circuits for sensory, social and repetitive behaviors, relevant to ASD, in a mammalian brain, and that some of these changes are reversible by SSRI retreatment.”

Pg4, ln19 “We developed a rodent model of maternal SSRI exposure, in the absence of maternal stress, to determine if drug alone induces behavioral disruptions related to the core symptoms of ASD in offspring.”

This is only when reaching pg19, ln8 that the reader is informed that Celf6 mutant mice are studied, together with heterozygous and wild-type littermates: “As we initially thought SSRI exposure alone might be a relatively modest factor, we exposed Celf6 mutant mice, which exhibit a subtle ASD-like phenotype (Dougherty et al., 2013), to maternal FLX and analyzed offspring behavior for possible potentiation of the ASD-like phenotype (Celf6-Extended).”

Because utilizing this mutant mouse line is crucial for segregating genetic vs environmental influences - this is the objective of the present study - it would be much more informative/correct if clearly stated in the abstract and introduction. In sum, I would suggest the authors to present the rationale of their study in a clearer way, without eluding the genetically vulnerable background for ASD, especially because this is a major strength of this study.

2) In the same line, I would suggest the authors to remain slightly more cautious when stating that “These findings indicate maternal fluoxetine treatment alone, independent of maternal stress, is sufficient to induce behavioral disruptions in mammalian offspring” (pg 1, ln16); that they “tested the hypothesis that maternal SSRI exposure alone, independent of maternal stress, is sufficient to modulate ASD-relevant behaviors in mammals” (pg30, ln11), simply because the potential impact of altered maternal behavior, especially altered maternal care, was not assessed. Is there any evidence that maternal care is unaffected in heterozygous Celf6 mothers (exposed, and not exposed to antidepressant)? Is there any evidence that antidepressant exposure alone, in wildtype mothers, has no impact on maternal care? Could it be that the combination of impaired early social communication (i.e. altered vocalization in Celf6-/- pups (see Dougherty 2013), potentially modulated/exacerbated by antidepressant exposure), and altered maternal care in heterozygous Celf6 mothers (potentially modulated/exacerbated by antidepressant exposure) does contribute to behavioral changes in the offspring, synergistically or not. I would suggest the authors to thoroughly discuss this issue, and remain slightly more cautious when interpreting their findings.

Minor:

- Fig6B & C: von Frey test, please correct “FILMAMENT FORCE”

## Author Response

Response to reviews, Maloney et al., MS No. eN-NWR-0120-18

Reviewers' comments:

Reviewer Comment 1: The statistical analyses appear for the most part to be correct. However there are a few concerns. First, if tests involve siblings from the same litter, one way to account for this factor is to include it as a covariate in any

of the models. Also for figures it is not clear why social preferences are expressed in box plots with medians and inter-

quartile ranges if they are parametric, then means SEM and SD would be far more conventional and also more clear for readers to understand.

Comment 1 Response: Litter as a variable is conflated with drug, so could not be used as a covariate. However, what we think the reviewer is referring to is litter size, and therefore we used litter size as a covariate and reanalyzed our data using Analyses of Covariance (ANCOVAs), where appropriate. We found only three instances out of almost 150 analyses in which the significant p value (&lt;.05) observed in the ANOVA was now non-significant (&gt;.05) when the means were adjusted for litter size in the ANCOVA. These include the P5 weight effect of drug for the long prenatal group (ANOVA: p=.043; ANCOVA: p=.059) and the P5 weight effect of drug for the short prenatal group (ANOVA: p=.023; ANCOVA: p=.304). The overall effect of drug for both cohorts on weight across P5, 7, 9, and 14 remained significant, as did the weight differences observed on P7, 9, and 14. Finally, the effect of drug on distance traveled during the chronic re- exposure testing period of the rescue cohort almost changed to marginally significant (ANOVA: p=.020; ANCOVA: p=.072). When directly compared, we only observed a significant difference in litter size between FLX and VEH litters in the short prenatal cohort. Thus, as very few analyses appeared to be influenced by litter size as a covariant, we feel confident in our use of chosen statistical tests and the interpretation of those results as independent of litter size.

We have now included additional text describing the ANCOVAs in the methods section (page 20), “As litter size can influence behavior, and our samples included littermates, we also conducted accompanying analyses of covariance (ANCOVAs) with litter size as the covariate, and report any discrepancies between the results.” We also reported the three altered results in the main text: (page 23) “The addition of litter size as a covariate in the model did not change the overall results of weight analyses for the three cohorts. However, the influence of drug on weights only at P5 for the

Long and Short Prenatal animals was found to be marginally significant (p=.059) and non-significant (p=.304) in the ANCOVA model.”; (page 31) “Analysis of covariance with litter size as the covariate yielded a marginally significant effect of drug on distance traveled during chronic re-exposure testing (p=.072).”

We appreciate the suggestion to convert the social approach social preferences figure into bar graphs to display means and SEMs. We also included all data points overlaid to display the entire distribution of the data. We feel this has improved and clarified Figure 3.

Reviewer Comment on Extended data:

Statistical tables are provided in text, they could alternately be placed in an appendix if more consistent with journal format.

Comment on Extended data Response: Per the advice from the reviewing editor, we kept the statistical tables in the text.

Reviewer Software comments:

1. Avisoft Recorder software for USV, with frequency sonograms prepared using MATLAB. No mention of validation of software.

2. MiceProfiler used for tracking both stimulus and experimental mice in tests of juvenile social interaction no validation but tracking was supervised and corrected manually, then MATLAB was used to process tracked videos. When frames were missing data points they were re-analyzed following smoothing.

3. Any-Maze software was used for social approach and 1-h locomotor activity, no validation tests or results were described.

Response to Software comments:

1. The methods for automated call acquisition and detection were based on previously published methods (Dougherty et al., 2013; Maloney et al., 2018; Rieger and Dougherty, 2016) adapted from a validated procedure (Holy and Guo,

2005). The pipeline includes manual inspection of random subsets of spectrograms (10-20% of files) to ensure the

automated scores overlap with human-distinguishable calls (Rieger and Dougherty, 2016). The initial procedure development included validation of the algorithm through performance comparisons with human inspection. Specifically: “The performance of this algorithm was compared with human inspection on 50 randomly selected 210-s trials. The algorithm successfully identified the vast majority (&gt;95%) of human-identified syllables, with systematic omission occurring only on the faintest and briefest syllables. False positives were encountered so rarely (two clear examples in 10,500 s of recording) that it was difficult to estimate their frequency, but they were clearly rarer than true syllables by several orders of magnitude. The algorithm also identified numerous vocalizations that were initially missed by a human observer, but which proved upon closer inspection to be correctly identified (verified graphically and by audible playback). The algorithm also identified the timing of the beginning and end of each syllable with high accuracy; occasional discrepancies with a human observer arose from interfering sounds or when the beginning or end of the syllable was unusually faint.”(Holy and Guo, 2005). We included the relevant citations in the methods section, “according to a previously published method (Dougherty et al., 2013; Maloney et al., 2018; Rieger and Dougherty, 2016), adapted from validated procedures (Holy and Guo, 2005).” (pg. 9)

2. MiceProfiler is a computerized tracking software that uses geometrical primitives to model and track two mice without requiring specific physical tagging, which may disrupt the social interaction. This software also allows for experimenter supervision of tracking through manual intervention and frame-by-frame correction. This method was previously published (de Chaumont et al., 2012) and validated by comparing results obtained with MiceProfiler to those obtained by human visual inspection. Social contact data was similar between supervised tracking with MiceProfiler and the experimenter-obtained values (de Chaumont et al., 2012). We highlighted this in our methods section on page 13, “This software allows for experimenter supervision of tracking through manual intervention and frame-by-frame correction, and was validated previously by comparing results obtained with MiceProfiler to those obtained by human visual inspection. Social contact data was similar between supervised tracking with MiceProfiler and the experimenter- obtained values (de Chaumont et al., 2012).”

3. ANY-maze tracking software is a commercially available software (Stoelting, Co.) that is used extensively in the rodent behavior literature for tracking movement throughout an arena (Dearborn et al., 2015; Dougherty et al., 2013; Martin et al., 2014; Miranda et al., 2015; Palanisamy et al., 2011; Rahn et al., 2012). An advantage of this software is that it allows for visual observation of the tracking being performed by highlighting the body and center point of the animal as it

tracks the movement through the arena. In addition, we visually screen the representation of the pathway traveled by each animal during testing to ensure no artifacts. If found, re-tracking was conducted of the video recording of the test. We added in our methods section the relevant citations that previously used this tracking software for social approach (Dougherty et al., 2013; Miranda et al., 2015) and 1-hr locomotor activity (Palanisamy et al., 2011).

The 1-hr locomotor activity task is a control measure to allow for proper interpretation of other behavioral tests that require a motoric response to ensure changes to activity levels are not driving any differences between groups. We did not observe differences in activity levels between our exposure groups. As these data are not central to the main findings of the manuscript, and as there is not supplementary sections permitted for the journal, after consultation with the editor it was decided to leave these data out of the main text.

Reviewers' Specific comments:

Overall a very interesting and potentially valuable manuscript examining in mice (wild-type and Celf6 mutant genotypes) the effects of 16 mg/kg/day fluoxetine exposure vs. vehicle during gestation with different stopping points for treatments on offspring behaviors relevant to autism. However since no measures of serum or brain fluoxetine or serotonin were made, it is not possible to know if the underlying mechanism is serotonin based. Further justification and explanation for

the value added from use of Celf6 mouse genotypes or relevance to serotonergic mechanisms underlying the behaviors is lacking. Some revisions, not necessarily requiring further experiments, are required to render this manuscript acceptable for publication. Specific revisions or clarifications are as follows:

Specific comment 1: Introduction: The rationale for use of the Celf6 mutation mice and their clinical relevance to autism is not provided until the results section, and it really needs to be introduced earlier in the manuscript. On the other hand a the “meta-analysis” information appears as two run-on sentences and doesn't draw the reader in. Maybe this analysis belongs in the discussion instead?

Response to specific comment 1: We moved up to the introduction the rationale for the gene x environment interaction component of the portion of the study examining the influence of FLX treatment on the genetically vulnerable background of Celf6 mutants. For details, please see our response to the Reviewers' Additional Comment 1 below.

We thank the reviewers for the editorial comment regarding the sentence describing the recent meta-analysis of epidemiological data. We have made this into two sentences (page 4), “A meta-analysis of the recent epidemiological studies examining this possible SSRI-ASD link reported a significant case-control association between maternal antidepressant use and ASD risk in offspring. This remained when adjusted for maternal psychiatric history (OR, 1.52;

95%CI, 1.09-2.12) (Mezzacappa et al., 2017), although parallel analysis of existing cohort studies did not quite show independence from psychiatric history (HR, 1.26; 95%CI, 0.91-1.74).” We left this in the introduction because we feel this background is important rationale for examining these behaviors in the rodent SSRI model.

Specific comment 2: In introduction, it is not clear what is meant by “acute deficits” and why they should be reversible, are the authors referring to 5-HT or SERT or behaviors?

Response to specific comment 2: What we meant here by “acute deficits” is that the effect (behavioral change) may be mediated by altered levels of 5-HT activity due to early FLX exposure, which are variable or adjustable through pharmacotherapy. This is a relatively more targetable mechanism in contrast to a permanent change in underlying cellular circuitry that is not amenable to fluctuations in 5-HT activity levels. To clarify this we changed the terminology to “persistent alterations in 5-HT activity levels”. Also, on page 37 we replaced the word “acute” with “abnormal” to clarify our meaning, “The partial rescue of the tactile hypersensitivity observed following adult FLX re-exposure suggests the circuit disruptions are reversible and may be due to abnormal 5-HT activity levels.”.

Specific comment 3: Abstract, introduction and throughout manuscript: for acronyms such as “SSRI” or “CNS” or “OCD” on page 25 etc...authors should spell out in entirety at first mention and introduce acronym.

Response to specific comment 3: We appreciate the editor and reviewers pointing out this missing information. We have defined each acronym at the first use.

Specific comment 4: Check spelling and grammar throughout, and proofread revision carefully to find mistakes: for example authors probably mean “trophic” not “tropic” in abstract, and introduction second paragraph “over” appears without context in middle of sentence.

Response to specific comment 4: We have proofread carefully to ensure proper spelling, grammar, and fixed any mistakes within the manuscript.

Specific comment 5: In the methods, was the Novalsan diluted at all, and if so by what %?

Response to specific comment 5: We used a 2% dilution of Nolvasan. We have included this information in the text.

Specific comment 6: Social dominance: Are three bouts per mouse sufficient to establish its dominance?

Response to specific comment 6: Two to three bouts in the tube test for social dominance is a standard procedure in the field (Korade et al., 2013; Lijam et al., 1997; Rodriguiz et al., 2004). Three bouts (against three different opponents) enables analysis of a percentage of wins vs loses that can be compared against what would be expected by chance, or

50%. The replication of the dominance phenotype in the FLX-exposed mice of the C57-Extended and Rescue cohorts provides a measure of reliability for our method in measuring dominant behavior.

Specific comment 7: What is the justification for the difference between “Long” and “Short” pre-natal? The distinction for this 4 day period seems arbitrary, so is there a basis for this distinction based on some critical biological change taking place during gestation?

Response to specific comment 7: The epidemiology studies examining the influence of SSRI exposure during pregnancy on risk of later ASD diagnosis show conflicting findings regarding which is the most vulnerable trimester. To address this issue of vulnerable periods in our mouse model, we chose to divide up the prenatal exposure into Short and Long because these mouse gestational periods correspond roughly in terms of brain development to the first trimester of human pregnancy (mouse E0-E16) and the majority of the second trimester (mouse E17-P0), and these distinguish distinct periods of serotonergic system development. It has been demonstrated that mouse embryonic stage at E7 is consistent with those in the human third week post-fertilization (Parnell et al., 2014; Sulik et al., 1981). Transition from the embryonic to fetal periods occurs during the beginning of the ninth week in human pregnancy and at approximately E14-E15 in the mouse (Otis and Brent, 1954; Parnell et al., 2014), and the overall morphology of the human and mouse brain is very similar during the transition between these developmental periods (Kaufman, 1994; Theiler, 1989). In addition, ethanol exposure during E0-E16 in the mouse produces the same morphological abnormalities and cognitive impairments observed during human pregnancy first trimester alcohol exposure that results in these abnormalities and impairments characteristic of fetal alcohol syndrome (Parnell et al., 2014). A distinction between mouse brain development before and after E17 is the development of the raphe system and the innervation of the forebrain by serotonergic fibers. The placental is the only source of serotonin to the developing forebrain until raphe innervation of the forebrain is complete at E18.5 (Muller et al., 2016). Therefore, exposure extending through this late gestational period in the mouse allowed for exposure to occur through this serotonergic innervation period. Thus, comparing short to long prenatal exposures allows for comparisons of FLX effects on the raphe nuclei development and, separately, its innervation of the forebrain.

Specific comment 8: The reduction in USV calls at postnatal days 5, 7, 9 measured appears to be the median number of calls per pup over 3 min. But it is not clear if same pups were measured repeatedly why these measures are not parametric. If parametric statistics were used then a repeated measures approach could be used. This measure appears to be highly variable, are outliers included or excluded from the analyses? Also given these differences, were fluoxetine exposed pups lost in the week following birth? There was some mention of smaller litter sizes in some fluoxetine treated groups.

Response to specific comment 8: We appreciate the opportunity to clarify these results. We analyzed the total number of USV calls during a 3 minute period across days 5, 7, and 9 using a repeated measures ANOVA (rmANOVA) with drug, and genotype if warranted, as between-subject factors. The same pups were measured each day, however, as was recently shown, USV call number is inconsistent across postnatal days within the same individuals, suggesting pup USV call number acts more like a state-like phenotype rather than a more stable trait-like phenotype (Rieger & Dougherty,

2016). We included any outliers (defined as z = {plus minus}3.5) in our final analyses because we found that exclusion of those data points did not impact the findings of the analysis and it was a better representation of the full data spectrum. We did

not observed an increased rate of pup mortality in the fluoxetine-exposed litters compared to the vehicle litters. We

observed a significant difference in litter sizes for the short prenatal cohort, in which the fluoxetine litters were on average smaller, yet we did not observe differences in USV call rate in this cohort.

Specific comment 9: Figure legends graphs and results text appear to be inconsistent with one another for adult social behavior and fig 3. For example, only figure D Celf6 -/- vehicle appear to have preference for interaction. While it states in the results that results are reported collapsed across sex, the fig 3 legend states a significant difference for female Celf6 mice after “D.” Also the authors report no significant differences, yet for E it appears that the vehicle treated mice might have a sociability preference, but this is not indicated by a star over bar.

Response to specific comment 9: We appreciate the reviewers alerting us to this. We removed the error in statistics reporting in the figure legend. All statistics are collapsed across sex as no interactions with sex were observed. We observed a significant preference for the social zone or the novel zone for all groups, except for long prenatal FLX- exposed animals during the sociability trial (Figure 3E). We represented these significant preferences through connecting lines above the cup zones bars with the actual p values in parentheses in lieu of asterisks to show significance. It may be that these values were obscured by the background colors. The vehicle-treated mice in 3E did indeed show a sociability preference, which was presented by the significant p value above the boxes. We hope that converting the graphs in this figure to bar graphs of means {plus minus} SEM and data points represented as circles clarifies this figure.

Specific comment 10: Results p. 25 marble bury paragraph for fig 5, what do the authors mean by “cryptic genetic differences” may be present? Could this due to errors in genotyping, and how reliable is the genotyping method being employed? Could other factors be involved, aside from maternal care such as sex of subjects?

Response to specific comment 10: We did not mean to imply any lack of confidence in the validity and reliability of our genotyping protocol: we use a standard genotyping master mix (https://www.neb.com/products/m0271-quick-load-taq-

2x-master-mix#Product%20Information) and primers that amplify a region spanning exons 3-4 (mutant allele lacks exon

4; as described in the Animals portion of Methods and Materials). We see an expected 152 base pair (bp) reduction in the mutant allele (mutant, 188 bp; wild type, 340 bp). We meant to imply that there may be an effect of background genome sequence (i.e. slight genetic drift due to random mutations) between inbred C57BL/6J mice from Jackson labs and the wild type littermates of the Celf6 heterozygous breedings (also on C57BL/6J background, but several generations removed). We do not think Sex is a likely influencing factor as an interaction with or main effect of Sex was

not observed in either the Celf6-Extended or C57-Extended cohorts. We substituted ‘subtle effects due to genetic drift in the Celf6 colony’ for ‘cryptic genetic differences’ to clarify our meaning (page 27).

Specific comment 11: For the independent Rescue cohort, were these mice also tested as the other mice had been during juvenile development? Also dubbing this group “retreatment” seems a bit off the mark, since first exposure is gestational as only the mother is treated, and second exposure is the actual treatment to correct deficits in offspring.

Response to specific comment 11: The mice in the Rescue cohort only received the test listed in Table 1, schematized in Figure 6 and listed in the Behavioral Tasks section in Methods and Materials, which include the von Frey assessment, T- maze, tube test, and 1-hr locomotor activity. We did not test these mice during juvenile development; testing began during early adulthood, ~P60. We added this sentence to the Behavioral Tasks section in Methods and Materials (page

10) to clarify this, “The Rescue cohort was not tested prior to re-exposure, such that no testing occurred during the pre-

weaning period or juvenile development.”

We agree “retreatment” may be a misnomer. We have replaced it throughout with “adult re-exposure” to indicate this

was distinct from maternal exposure.

Specific comment 12: The study lacks biochemical measures that could provide mechanistic insights. Were any measures of fluoxetine taken from maternal blood or brain or pup blood or brain to determine long term exposure levels?

Response to specific comment 12: Given the half-life of fluoxetine (FLX; ~6h) and its active metabolite norfluoxetine (NFLX; ~15 h) in vivo, both should be well cleared by the time the juvenile and adult offspring were analyzed. However, we shared the reviewers' interest in whether the early postnatal time points might be influenced by ongoing FLX/NFLX in the brain. Therefore, we worked in collaboration with researchers at Wright State University, Drs. Audrey McGowin and Adrian Corbett(Corbett et al., 2012) to acquire FLX and NFLX levels via HPLC from the brains of dams and pups. This experiment yielded a clear result from FLX- and vehicle-exposed dams and extended exposure pups at P9 (See Table 3 in text): it confirms the drug is present in the P9 brains.

We have incorporated the data from our experiment into the manuscript. The results can be found on page 24 and the data in Table 3, “To confirm the drug was reaching the developing brain, HPLC was used to measure levels of FLX and its active metabolite NFLX in whole brains of pups exposed to extended maternal FLX exposure. We found FLX and NFLX were both present in the P9 pup brain during maternal FLX exposure, and neither present in the VEH-exposed control brains (Table 3). The levels of FLX and NFLX in the pups were about 43% and 32%, respectively, of that measured in an equal amount of dam brain tissue. These data indicate that FLX and NFLX are active in the offspring brain during

maternal exposure, suggesting the 5-HT system is targeted at this time. Given the half-life of fluoxetine (FLX; ~6h) and its active metabolite norfluoxetine (NFLX; ~15 h) in vivo, both should be well cleared by juvenile and adult ages (Holladay et al., 1998; Marken and Munro, 2000). Thus while the alterations in USV behavior might be impacted by the acute levels

of FLX and NFLX, the later behavioral alterations must reflect long term consequences of transient exposure.”

We also included a description of HPLC methods on pages 8-9, and added Drs. Audrey McGowin and Adrian Corbett as authors for their contributions to this work.

Specific comment 13: In discussion the authors describe the study as having a “well-powered manner”, but specifically what they mean by this is not clear.

Response to specific comment 13: The term “well-powered manner” was used to describe our effort to utilize samples sizes at or above 20 individuals per group for between-drug comparisons. A sample size of 26 is required with a large effect size of f=0.40 and 80% power to reject the null hypothesis that two groups are not equal. Any smaller effect would thus require even more animals. Therefore, our goal was to achieve sample sizes per exposure group within each cohort as close to that as possible, with equal distributed between males and females, to feel confident in our interpretation of the findings. However, as inclusion of that term does not seem to convey our attempted meaning, we have removed it from the discussion.

Specific comment 14: In references some of the titles are capitalized on all words while others only on first word, choose the format consistent with the journal and make sure it is used throughout.

Response to specific comment 14: This inconsistency was due to differences in importation styles into our Zotero bibliography software. We fixed the capitalization inconsistencies within the citations and the references list is now consistent.

Reviewers' additional specific comments:

Additional comment 1: In the abstract, significance statement and introduction sections (and title), the authors do not clearly mention that the experiments were carried out in mice with a genetically vulnerable background for autism

spectrum disorders (i.e. Celf6-/- mice).

Pg1, ln6: “Here, we sought to determine if maternal SSRI treatment alone, independent of maternal stress, as sufficient

to induce in the offspring behavior disruptions relevant to ASD.”

Pg, ln5: “Our mouse studies show that, in the absence of other maternal manipulations or stressors, developmental SSRI exposure alone can alter the long-term functioning of behavioral circuits for sensory, social and repetitive behaviors, relevant to ASD, in a mammalian brain, and that some of these changes are reversible by SSRI retreatment.”

Pg4, ln19 “We developed a rodent model of maternal SSRI exposure, in the absence of maternal stress, to determine if

drug alone induces behavioral disruptions related to the core symptoms of ASD in offspring.”

This is only when reaching pg19, ln8 that the reader is informed that Celf6 mutant mice are studied, together with heterozygous and wild-type littermates: “As we initially thought SSRI exposure alone might be a relatively modest factor, we exposed Celf6 mutant mice, which exhibit a subtle ASD-like phenotype (Dougherty et al., 2013), to maternal FLX and analyzed offspring behavior for possible potentiation of the ASD-like phenotype (Celf6-Extended).”

Because utilizing this mutant mouse line is crucial for segregating genetic vs environmental influences - this is the objective of the present study - it would be much more informative/correct if clearly stated in the abstract and introduction. In sum, I would suggest the authors to present the rationale of their study in a clearer way, without eluding the genetically vulnerable background for ASD, especially because this is a major strength of this study.

Response to additional comment 1: We appreciate this comment and agree that the rationale for the Celf6 x SSRI interaction component of the manuscript needs to be appropriately introduced/described. To achieve this, we have made the changes below in red text to the following statements:

Pg1, ln6: “Here, we sought to determine if maternal SSRI treatment alone, independent of maternal stress, as sufficient to induce in the offspring behavior disruptions relevant to ASD.”

New sentence: Here, we sought to determine if maternal SSRI treatment alone or in combination with a genetically vulnerable background, independent of maternal stress, was sufficient to induce in the offspring behavior disruptions relevant to ASD.

We also included several lines later in the Abstract, “Celf6 mutant mice demonstrate social communicative and perseverative behaviors, yet an interaction with maternal FLX was not observed.”

Pg, ln5: “Our mouse studies show that, in the absence of other maternal manipulations or stressors, developmental SSRI exposure alone can alter the long-term functioning of behavioral circuits for sensory, social and repetitive behaviors, relevant to ASD, in a mammalian brain, and that some of these changes are reversible by SSRI retreatment.”

Response: We did not think it appropriate to alter this sentence to include the Celf6 mutant study because we did not find a direct interaction between the Celf6 mutation and exposure to maternal FLX. Thus, we can really only make the statement that SSRI alone, not in combination with the vulnerable background used in this study, can alter long-term functioning.

Pg4, ln19 “We developed a rodent model of maternal SSRI exposure, in the absence of maternal stress, to determine if drug alone induces behavioral disruptions related to the core symptoms of ASD in offspring.”

We moved our rationale for use of the Celf6 mutant line from the results section up to the introduction, and placed it directly following the above sentence:

As genetic factors are clearly an important causation of ASD (Geschwind, 2008), it is likely that environmental

contributions to ASD risk interact with existing genetic susceptibility (Hertz-Picciotto et al., 2006; Klei et al., 2012). It has been suggested that environmental factors that might modulate social behavior or language could tip the balance towards ASD in children with genetic vulnerability (Geschwind, 2008). As we initially thought SSRI exposure alone might be a relatively modest factor, we also exposed Celf6 mutant mice, which exhibit a subtle ASD-like phenotype (Dougherty et al., 2013), to maternal SSRI and analyzed offspring behavior for possible potentiation of the ASD-like phenotype. The

Celf6 mutant was ideal for this gene x environment experiment because this model already shows subtle ASD-related deficits, specifically decreased early social communicative behavior and a resistance to change behavior patterns (Dougherty et al., 2013), which allows for possible further disruption to other social and repetitive behaviors with the addition of FLX. Further, Celf6 is enriched in 5-HT-producing cells and, when deleted, results in a decrease in brain 5-HT levels (Dougherty et al., 2013). Thus, we hypothesized that early exposure to FLX may interact synergistically on the 5-HT system to further disrupt behavior in mice with this genetically vulnerable background.

Additional comment 2: In the same line, I would suggest the authors to remain slightly more cautious when stating that “These findings indicate maternal fluoxetine treatment alone, independent of maternal stress, is sufficient to induce behavioral disruptions in mammalian offspring” (pg 1, ln16); that they “tested the hypothesis that maternal SSRI exposure alone, independent of maternal stress, is sufficient to modulate ASD-relevant behaviors in mammals” (pg30, ln11), simply because the potential impact of altered maternal behavior, especially altered maternal care, was not assessed. Is there any evidence that maternal care is unaffected in heterozygous Celf6 mothers (exposed, and not exposed to antidepressant)? Is there any evidence that antidepressant exposure alone, in wildtype mothers, has no impact on maternal care? Could it be that the combination of impaired early social communication (i.e. altered vocalization in Celf6-/- pups (see Dougherty 2013), potentially modulated/exacerbated by antidepressant exposure), and altered maternal care in heterozygous Celf6 mothers (potentially modulated/exacerbated by antidepressant exposure) does contribute to behavioral changes in the offspring, synergistically or not. I would suggest the authors to thoroughly discuss this issue, and remain slightly more cautious when interpreting their findings.

Response to additional comment 2: We toned down our concluding statements to now read, “These findings indicate maternal fluoxetine treatment, independent of maternal stress, can induce behavioral disruptions in mammalian offspring” and “we tested the hypothesis that maternal SSRI exposure, independent of maternal stress, can modulate ASD-relevant behaviors in mammals.”

We further discussed the issue of potentially altered maternal care in our discussion on page 38-39, “The potential influence of maternal care is complex, and worthy of an entire study of its own. Qualitatively, differences in maternal care have not been observed in Celf6+/- dams, yet this has not been thoroughly quantified. In addition, there may be an interaction between direct FLX exposure and heterozygous loss of Celf6 that affects maternal behavior and maternal care. The reciprocal influence of maternal care and pup USV on each other is complex. Greater maternal responsiveness has been shown to result in fewer calls emitted by the pups (D'Amato et al., 2005). However, decreased USV production by pups has also been shown to result in maternal neglect because the dams cannot locate the pups outside of the nest. This was identified in vocally impaired pups with genetic loss of motor neurons that transform breaths into calls (Hernandez-Miranda et al., 2017). It is possible in our FLX model that pup USV and maternal care are interacting in several ways. First, FLX could be directly impacting maternal care, and decreasing pup USVs. If this is the case in our mice, we would hypothesize based on previous research that the FLX increased maternal care and thus reduced pup USVs. We would further hypothesize this level of maternal care would likely not result in the long-term behavioral deficits observed in the adult offspring. However, the large magnitude of the reduction in USV we observed in FLX-

exposed pups seems too robust for changes in maternal care to account for the underlying the pup phenotype. A second way in which maternal care and pup USV may be interacting is through a reduction to maternal care in response to the robustly reduced USV emitted by the pups exposed to FLX. This reduced maternal care has the potential to further disrupt neurodevelopment of the pup, and thus be a possible indirect influence on the later adult behaviors. Celf6 mutation harbored by the dam may also play into this scenario by altering dam or pup responses additively or synergistically. To our knowledge, the direct impact of SSRI exposure on maternal behaviors has not been examined; however increased latency to retrieve pups back to the nest has been demonstrated in adult female offspring exposed gestationally to FLX (Svirsky et al., 2016), suggesting trans-generational effects of gestational FLX exposure. Thus, we can conclude that FLX treatment to the dam during and immediately following pregnancy modulates progeny behaviors relevant to ASD; and that this is independent of maternal stress but possibly mediated by alterations to maternal care behaviors.”

Reviewers' Minor Comment:

- Fig6B & C: von Frey test, please correct “FILMAMENT FORCE”

Response to minor comment:

We have corrected this typo in our figure and appreciate the reviewers flagging it for us.

We appreciate the comments provided by the editor and reviewers and believe these revisions strengthen our manuscript.
